# Engineered Membrane Vesicle Production via *oprF* or *oprI* Deletion Has Distinct Phenotypic Effects in *Pseudomonas putida*

**DOI:** 10.1021/acssynbio.5c00171

**Published:** 2025-07-03

**Authors:** Rebecca A. Wilkes, Tarryn E. Miller, Jacob Waldbauer, Nanqing Zhou, Lichun Zhang, Beth N. DiBiase, Neha P. Kamat, Ludmilla Aristilde, Gregg T. Beckham, Allison Z. Werner

**Affiliations:** † Renewable Resources and Enabling Sciences Center, 53405 National Renewable Energy Laboratory, Golden, Colorado 80401-3393, United States; ‡ Department of the Geophysical Sciences, 2462 University of Chicago, Chicago, Illinois 60637-5418, United States; § Department of Civil and Environmental Engineering, McCormick School of Engineering and Applied Science, 3270 Northwestern University, Evanston, Illinois 60208-0001, United States; ∥ Department of Chemical and Biological Engineering, 3270 Northwestern University, Evanston, Illinois 60208-0001, United States; ⊥ Department of Biomedical Engineering, 3270 Northwestern University, Evanston, Illinois 60208-0001, United States

**Keywords:** membrane vesicles, outer membrane vesicles, hypervesiculation, proteomics, protein secretion

## Abstract

Membrane vesicle (MV) production is a natural phenomenon in Gram-negative bacteria and represents an emerging synthetic biology tool for the secretion of biomolecules or bioproducts. Manipulation of membrane components has proven successful in enhancing MV production. However, the impact of membrane disruptions on strain fitness and protein composition warrants further investigation for the use of MVs in industrial bioprocesses. Here, we identify and characterize two genetic engineering strategies for inducing hypervesiculation—deletion of genes for the outer membrane porin OprF or the lipoprotein OprI—in the commonly used platform KT2440. Deletion of *oprI* generated up to a 1.5-fold increase in MVs, larger MVs with a greater proportion of outer membrane proteins, and no significant impact on strain fitness compared to wild type. In contrast, deletion of *oprF,* relative to wild type, generated up to a 4-fold increase in MVs but diminished growth, permeabilized membranes, and increased cytosolic protein packaging. Both hypervesiculation phenotypes increased nontargeted and MV-targeted mNeonGreen extracellular signal by up to 6-fold, demonstrating vesiculation as a mechanism for protein secretion. Despite increased blebbing of MVs from gene deletions, proteins involved in membrane biosynthesis were not elevated relative to wild type. Overexpression of *gpsA,* which initiates glycerophospholipid biosynthesis, in the Δ*oprF* background improved the membrane integrity by 37% and maintained MV formation, highlighting the importance of membrane biosynthesis in restoring the membrane in hypervesiculating strains. Together, this study provides genetic engineering strategies with corresponding phenotypic outcomes toward providing a synthetic biology toolset for MV deployment in .

## Graphical abstract

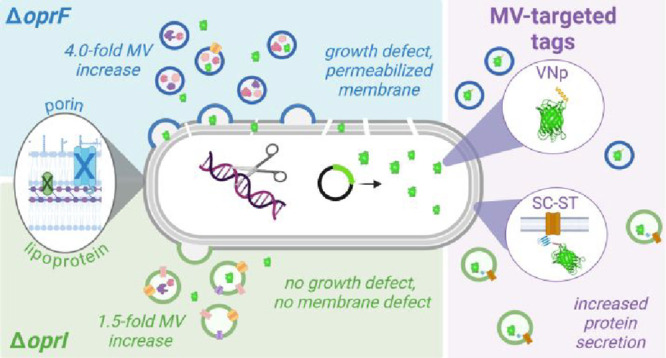

## Introduction

Gram-negative bacteria generate membrane vesicles (MVs) by blebbing portions of the membrane to create spherical lipid bilayers encircling cellular constituents, including proteins and nucleic acids.^1,2^ MVs have a myriad of proposed functions, such as removing toxic compounds or misfolded proteins, improving resistance to chemical stressors, scavenging of essential nutrients (i.e., carbon, nitrogen, or metals), exchanging genetic material, and defending against phages.^2–4^ Due to their role in diverse cellular functions and ability to encapsulate biological molecules, MVs have gained attention as versatile tools for bioproduction, biosensing, drug delivery, and vaccine development, among other applications.^4–6^

As knowledge of MV formation and packaging has grown, biotechnological applications of MVs have expanded to include secretion mechanisms for hydrophobic chemicals and proteins,^7–11^ lipid protectors of enzymes,^12–15^ detection systems of biomolecules,^16,17^ cell-free remediators of environmental pollutants,^13,18^ and scaffolds for coordinating enzymatic reactions.^19,20^ These diverse applications depend on genetic engineering strategies to increase vesicle formation, also called hypervesiculation, and target macromolecules to MVs.

Various strategies exist to induce vesiculation in Gram-negative bacteria. Vesiculation has been triggered through exposure to a stress stimulus, such as *Pseudomonas* quinolone signal (PQS) or 1-octanol.^9,21,22^ Genetic engineering efforts have included both deletion and overexpression of key proteins that either destabilize the outer membrane linkages to the peptidoglycan layer, accumulate proteins or peptidoglycan fragments in the periplasm to generate turgor pressure on the outer membrane, or alter the composition of the phospholipids in the outer membrane to increase curvature.^23–26^ To target proteins for encapsulation in or display on MVs, previous work in has exploited outer membrane proteins as anchors for enzymes of interest using a direct fusion linkage or SpyCatcher-SpyTag (SC-ST) bioconjugation system.^12,16,19,20,27^ Without a protein anchor, vesicle nucleating peptide (VNp) and pectate lyase B were used to increase the associations of recombinant proteins with MVs in .^28,29^ The combination of tools to trigger hypervesiculation and target proteins to MVs has the potential to improve the export and packaging of proteins.

 KT2440 (hereafter KT2440) is a Gram-negative bacterium noted for its catabolic versatility, stress tolerance, and genetic tractability.^30–34^ In particular, KT2440 has been demonstrated previously to tolerate and catabolize complex feedstocks containing high concentrations of aromatic and aliphatic acids such as lignin and plastic streams.^35–42^ Notably, alkaline pretreated liquor (APL) from corn stover, a lignin stream rich in aromatic compounds, increased MV blebbing for KT2440 relative to being grown on glucose alone.^43^ Further, the MVs isolated during growth with APL or on benzoate alone were found to contain enzymes belonging to the aromatic cleavage pathways,^43,44^ implying their potential for aromatic carbon turnover. Under different temperature, osmotic, and toxin stress conditions, a different strain, DOT-T1E, released MVs resulting in an increase in cell surface hydrophobicity and a tendency to form biofilms, highlighting the role of MVs as a stress response in .^45^ Concurrent to this work, Bitzenhofer and colleagues found that down- or up-regulation of a subset of genes in KT2440 using a CRISPRi system induced vesiculation, and used this as a strategy to increase natural product yields.^9^ For widespread deployment of MVs as a synthetic biology tool in , a greater understanding of the impact of genetic manipulation to create a hypervesiculation phenotype on cellular fitness and the MV protein cargo is warranted.

In this work, we aimed to establish a genetic toolkit for hypervesiculation in KT2440 and characterize the impact on growth phenotype, membrane permeability, protein composition, and protein export. We identified two strategies that increased vesiculation relative to the wild-type strain: deletion of the genes encoding either the outer membrane porin OprF or the lipoprotein OprI. We determined that Δ*oprF* displayed the stronger hypervesiculation phenotype yet had a greater deleterious impact on cellular growth and membrane integrity. Next, we found that hypervesiculation improved protein export, regardless of whether it was targeted to MVs with SC-ST bioconjugation or VNp tags. Lastly, we examined the impact of overexpressing genes involved in membrane biosynthesis in Δ*oprF* as a method to restore membrane integrity or increase vesiculation further. Overall, this work provides genetic strategies alongside physiological characterization for hypervesiculation in KT2440, laying the foundation for engineering MV formation and protein export that can be employed for various biotechnological applications.

## Results and Discussion

### Deletion of Genes for the Outer Membrane Porin OprF or Lipoprotein OprI Increased MV Production

In Gram-negative bacteria, genetic manipulations of genes associated with the outer membrane and peptidoglycan layers have been found to induce vesiculation.^9,11,23,25,26,46–48^ Here, we first compared genetic strategies in KT2440 that were predicted to either (i) destabilize the outer membrane resulting in MV blebbing or (ii) accumulate phospholipids in the outer leaflet resulting in increased curvature of the outer membrane (Figure 1A and Table S1). From these targets, nine candidate gene knockouts were constructed, including six porins with 34–42% identity to OmpA in (Table S2), one lipoprotein, and two proteins with roles in the Mla anterograde phospholipid shuttling pathway (Figure 1A and Table S1). Gene knockouts in KT2440 were obtained via markerless gene deletions or from an arrayed randomly barcoded transposon (Tn) mutant library (Table S3).^49,50^ Despite several attempts, clean deletions of *tolA*, *tolB*, *tolR*, or *pal* (also called *oprL*) could not be generated nor were they obtained in the Tn mutant library, suggesting the essentiality of these genes to KT2440. However, unlike and ,^29,51,52^ knockouts of the Tol-Pal complex are likely not suitable targets for hypervesiculation in KT2440 because knocking down *tolA* or *pal* using a CRISPRi system did not significantly increase the MV formation.^9^ In addition to the constructed genetic knockouts, supplementing the media with the external stimulus PQS was evaluated as a control, as this has been shown to increase MV production in by intercalating into the outer membrane to increase curvature (Figure 1A).^21^

**Figure 1:**
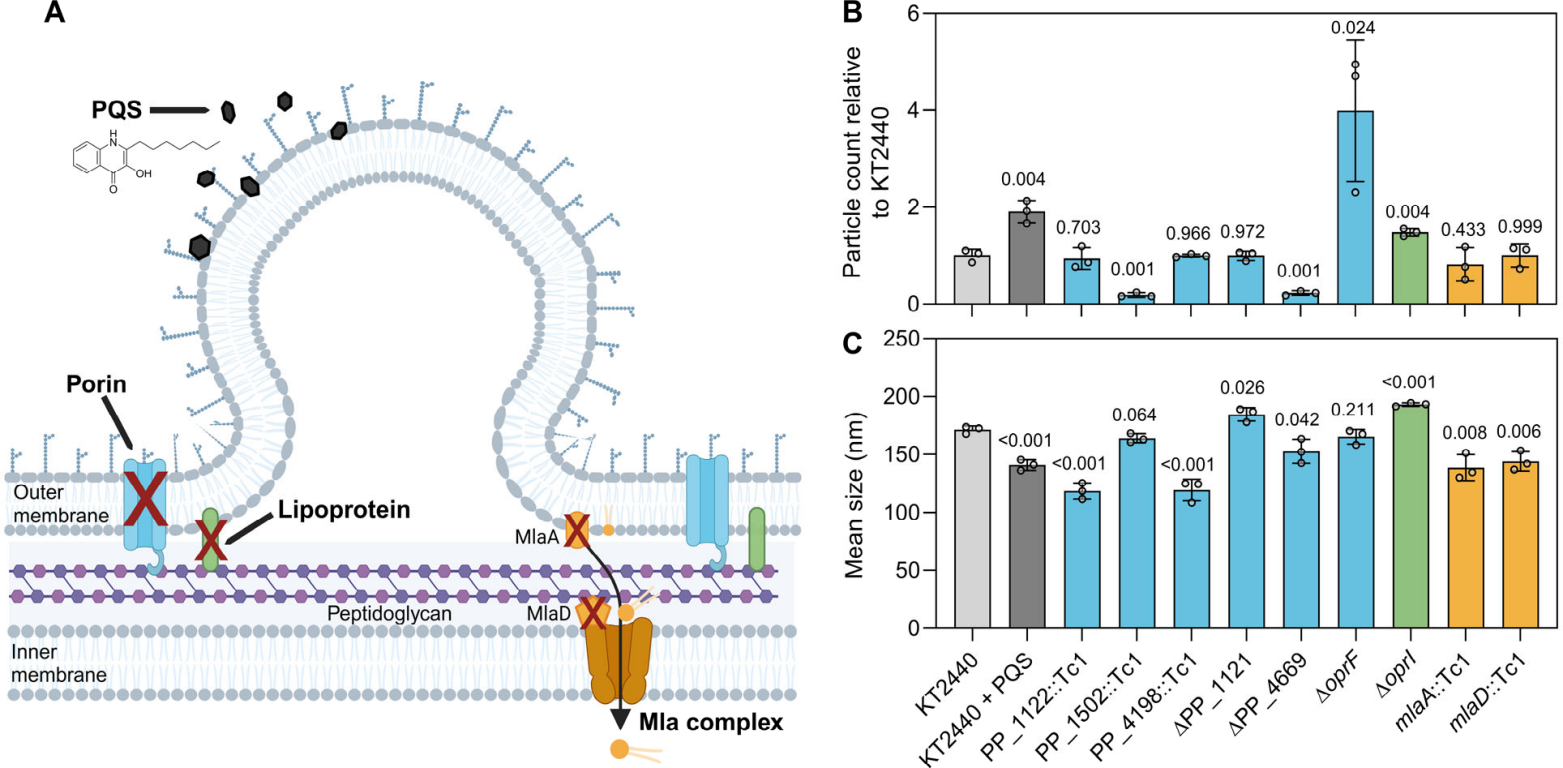
MV particle counts and sizes for genetic mutants of outer membrane porin, lipoprotein, and phospholipid-shuttling genes. (A) Schematic of mechanisms to engineer hypervesiculation. Created in BioRender.com. (B) Particle counts were measured in the extracted MV fraction and normalized by g_CDW_ for the knockout strains relative to the average of the triplicate extractions for KT2440 (WT). The particles per g_CDW_ values are provided in Figure S1 and the absolute counts are provided in Excel file 1. As a positive control for hypervesiculation,^9^ KT2440 was exposed to 50 μM *Pseudomonas* quinolone signal (PQS). (C) Mean size of particle diameters (nm) measured for KT2440 and all knockout strains. For B and C, the data represent the mean ± the standard deviation determined from three biological replicates. Individual points are illustrated for each biological replicate. Significant differences (*p* < 0.05) were determined with an unpaired two-tailed *t*-test between the engineered strains and wild-type KT2440.

To compare the impact of these strategies on MV production in KT2440, the nine strains were grown to late-exponential phase on 20 mM glucose before harvesting the MV fraction from the clarified supernatant using affinity columns, as described previously.^43^ Particle counts and size distributions of MVs were determined using nanoparticle tracking analysis (NTA) on samples that had only undergone one freeze–thaw cycle. MV counts normalized by the gram cell dry weight (g_CDW_) and MV sizes were compared to wild-type KT2440 (Figures 1B, S1 and Excel file 1). Differences in MV concentration were confirmed to have no impact on the OD_600_ measurements obtained to monitor growth (Figure S2).

The addition of 50 μM PQS at the start of growth led to a 1.9-fold increase in the MV particle count, supporting that this external stimulus can be utilized to trigger vesiculation in more species than just , which natively produces the PQS.^9,21,22^ Across the six OmpA-like proteins evaluated, only Δ*oprF* increased particle counts relative to KT2440, which was the highest increase across tested strains at 4.0-fold (Figure 1B). Additionally, deletion of *oprI,* which encodes a lipoprotein, increased the particle count by 1.5-fold (Figure 1B). In contrast to Δ*oprF*, which did not significantly alter the mean particle size relative to KT2440, Δ*oprI* increased the mean particle size by 22 nm, indicating that there were potential differences in MV formation across genetic strategies (Figure 1C).

The *mlaA* and *mlaD* Tn insertion mutants did not alter the MVs counts in KT2440, different from observations for Δ*mlaA* in or Δ*vacJ* (an MlaA homologue) in and (Figure 1B).^11,25^ This is consistent with prior work that found knocking down *mlaE* was not a leading strategy for hypervesiculation in KT2440.^9^ Notably, as hypovesiculation phenotypes are not as commonly reported,^9,25,53^ approximately 5-fold decreases in particle counts were observed for two putative porins, the PP_1502 Tn insertion mutant and ΔPP_4669 (Figure 1B). This hypovesiculation phenotype was not observed in a clean deletion of ΔPP_1502 but was observed repeatedly for ΔPP_4669 (Figure S3). Among the genetic knockouts that did not increase vesicle formation, there were changes in the mean size of the particles produced relative to KT2440 (Figure 1C), implying that although these candidate genes did not increase the total number of MVs, they may still play a role in the cellular mechanisms behind blebbing (Figure 1C). Overall, only Δ*oprF* and Δ*oprI* increased and only ΔPP_4669 decreased significantly the particle counts relative to wild-type KT2440, and thus, these three strains were pursued further to understand the impact of genetically altered vesiculation on cellular growth and MV phenotypes.

### Deletion of *oprF* Decreased Tolerance to HCAs and Increased Membrane Permeability

We next evaluated how the top hypervesiculation strategies–deletion of *oprF* encoding an outer membrane porin or *oprI* encoding a lipoprotein–impacted cellular growth. Deletion of the gene encoding the OmpA family protein PP_4669 was included to assess the hypovesiculation phenotype and as a control for the deletion of a gene encoding an outer membrane protein that did not result in increased vesicle formation. Glucose and the lignin-related hydroxycinnamic acids (HCAs) *p-*coumarate and ferulate, relevant to bioprocessing of lignocellulosic waste streams, were used as substrates because aromatic compounds can impact growth and interfere with membrane structure and integrity.^42,54,55^ Thus, the combination of membrane instability from deletion of an outer membrane protein could be synergistic with HCA toxicity and result in diminished growth. To assess this, lag phase, growth rate, and membrane permeability were evaluated on 20 mM glucose alone or with different concentrations of *p*-coumarate and/or ferulate (Figure 2A–C).

**Figure 2:**
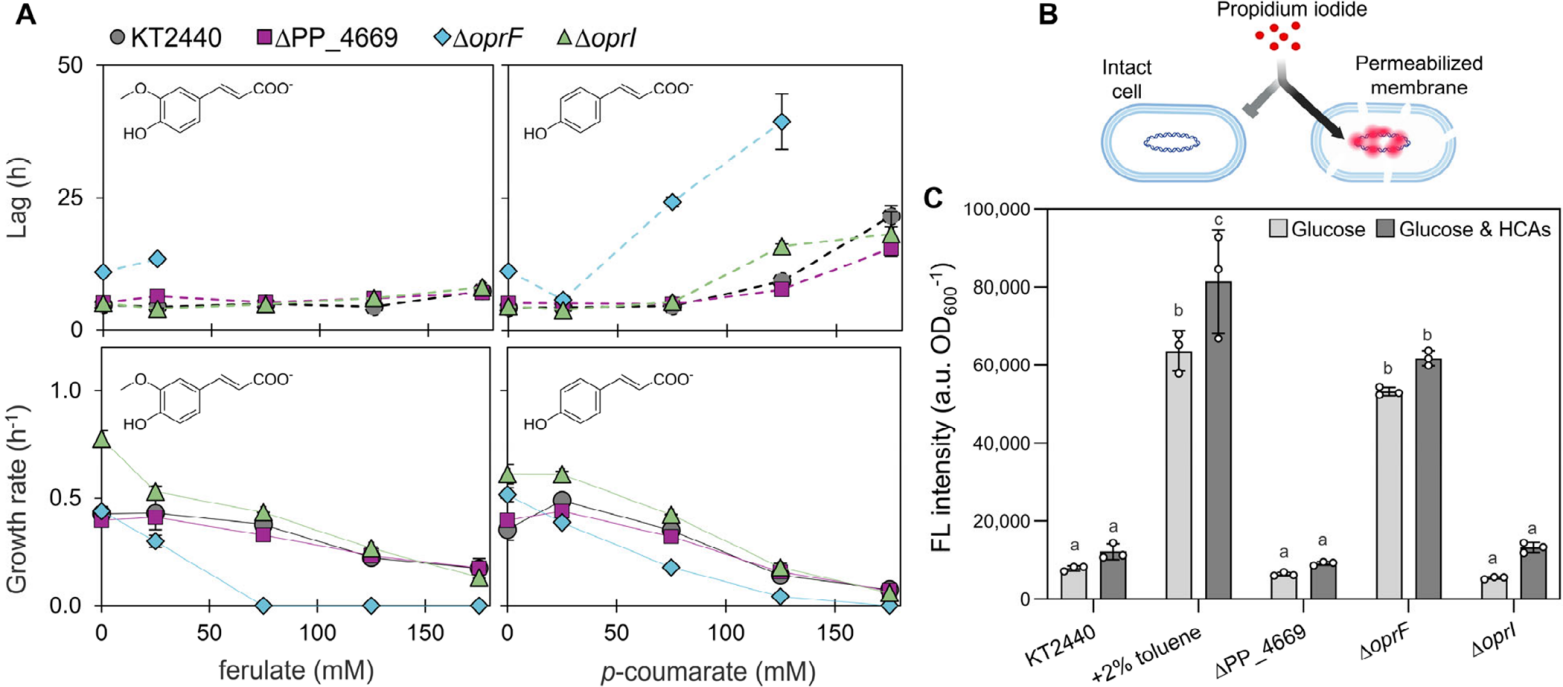
Lag phase, growth rate, and membrane permeability in *oprF, oprI*, and PP_4669 deletion mutants during growth on HCAs. (A) Growth rate and lag of wild-type KT2440 and the three deletion mutants on 20 mM glucose plus increasing concentrations of *p*-coumarate or ferulate. Cells were grown in 96-well plates and maximum specific growth rates and lags were calculated using Gompertz fits.^56^ Growth dynamics data is provided in Excel file 1. (B) Schematic of membrane integrity assay illustrating propidium iodide binding to nucleic acids when passing through a permeabilized membrane. Created in BioRender.com. (C) Fluorescence (FL) measurements indicating membrane permeability of the strains after growth on 20 mM glucose alone or 20 mM glucose plus HCAs (12.5 mM *p*-coumarate and 12.5 mM ferulate) until an OD_600_ of 0.9–1.2. As a positive control for permeable cells, KT2440 was exposed to 2% toluene for 30 min. Statistically significant differences (*p* < 0.05) determined by one-way ANOVA followed by Tukey HSD post hoc tests are denoted by a change in letter. Exact *p-*values and all raw data are provided in Excel file 1.

On glucose alone, the lag phase observed in the Δ*oprF* cultivations increased by 6.4 h whereas Δ*oprI* and ΔPP_4669 did not significantly alter growth compared to KT2440 (Figure 2A). In the presence of HCAs, Δ*oprF* had substantially diminished growth, with a 10 h increase in lag phase and a 26% decrease in growth rate compared to KT2440 at 25 mM ferulate and no growth was observed at 75 mM ferulate or 175 mM *p*-coumarate (Figure 2A). Thus, deletion of *oprF* but not *oprI* resulted in substantial growth defects on HCAs, indicating a difference in the extent to which the lack of these proteins destabilizes the outer membrane to peptidoglycan linkages. Such a destabilization could both trigger MV blebbing (Figure 1) and increase membrane permeability, which could have broad impacts on cellular fitness.

To assess the membrane permeability, propidium iodide binding to nucleic acids was assayed, as described previously (Figure 2B).^57^ Membrane permeability of Δ*oprF,* Δ*oprI,* and ΔPP_4669 were compared to KT2440 during growth on 20 mM glucose alone or a mixture of 20 mM glucose plus 12.5 mM *p-*coumarate and 12.5 mM ferulate (Figure 2C) as Δ*oprF* growth was impacted at 25 mM or higher concentrations of both ferulate and *p*-coumarate (Figure 2A). As a positive control for permeabilized membranes, wild-type cells were exposed to 2% toluene for 30 min, which resulted in an 8.1-fold and 5.8-fold increase, respectively, after growth on glucose and glucose plus HCAs. On either substrate composition, there was no significant difference in the membrane integrity of Δ*oprI* or ΔPP_4669 relative to KT2440. However, Δ*oprF* had a 6.7-fold and 4.3-fold increase in fluorescence intensity when grown on glucose or glucose plus HCAs compared to KT2440 (Figure 2C). Taken together, although Δ*oprF* displayed the highest MV production, it also displayed lower tolerance to HCAs, which we attributed, in part, to the compromised membrane integrity of this strain. Impaired growth and tolerance would be detrimental to the usage of this hypervesiculation strain for expanded substrate utilization or bioproduction of chemicals. Conversely, growth and membrane permeability are largely unaffected for Δ*oprI*.

### Aromatic Catabolism Is Altered in Hypervesiculation Strains

We next evaluated whether Δ*oprF* and Δ*oprI* strategies impacted aromatic compound catabolism and sustained the hypervesiculation phenotypes during growth on HCAs, again using hypovesiculating ΔPP_4669 for comparison. Strains were cultivated on a mixture of 20 mM glucose, 12.5 mM *p-*coumarate, and 12.5 mM ferulate. Extracellular concentrations of substrates and aromatic pathway intermediates were quantified over time (Figure 3A,B). MVs were harvested when cells were at an OD_600_ of 1.3–1.7, corresponding to exponential growth and depletion of all three substrates, and analyzed by NTA as described above.

**Figure 3:**
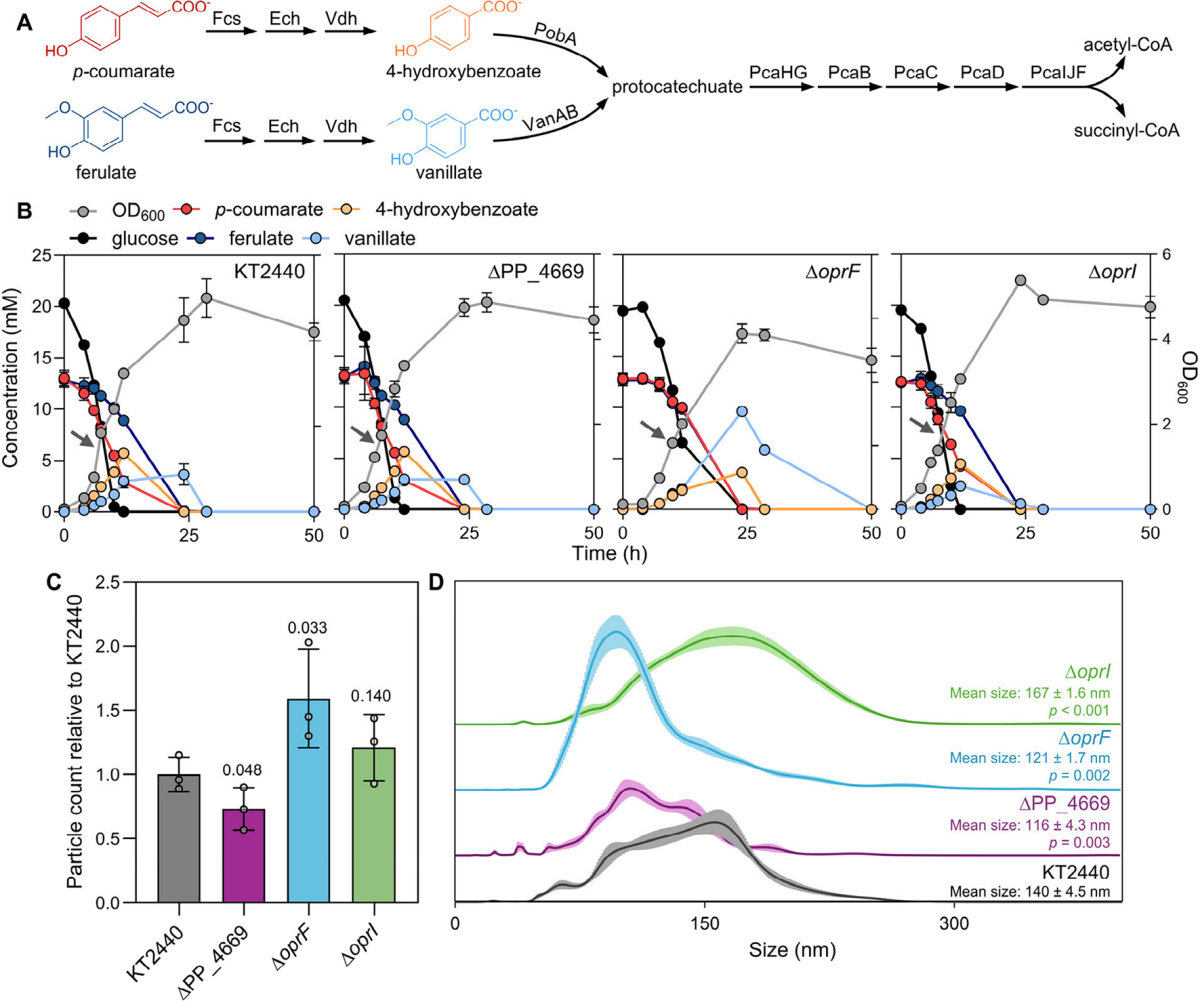
HCA catabolism and MV production by *oprI*, *oprF*, and PP_4669 mutants. (A) Schematic of *p-*coumarate and ferulate catabolism pathway in KT2440 with intermediates found in extracellular milieu highlighted. (B) Growth and consumption dynamics of KT2440 and knockout strains grown on 20 mM glucose plus 12.5 mM *p-*coumarate and 12.5 mM ferulate. The measured concentrations are also provided in Excel file 1. The arrows indicate the sampling point for MV extractions. Error bars represent the standard deviation of the average of three biological replicates. (C) Particle counts normalized to g_CDW_ for the deletion strains relative to the average of the triplicate counts for KT2440. The particles per g_CDW_ values are provided in Figure S4 and the absolute counts are provided in Excel file 1. Individual points are shown for each of the biological triplicates; error bars represent standard deviation across measurements. (D) The size distribution of the MVs was measured using NTA for the KT2440 and knockout strains. The line represents the mean and the shaded area indicates the standard deviation of three biological replicates. The average mean size ± standard deviation of the three biological replicates is listed. For C and D, significant differences (*p* < 0.05) were calculated, respectively, using unpaired one-tailed *t*-test for particle count and two-tailed *t*-test for the mean size.

Wild-type KT2440 cultivations simultaneously catabolized all three substrates and transiently accumulated 4-hydroxybenzoate and vanillate catabolic intermediates (Figure 3B). Deletion of *oprI* reduced vanillate accumulation by 3.05 mM at 24 h as compared to wild type. Conversely, deletion of *oprF* increased vanillate accumulation by 6 mM at 24 h, along with a 4 h increase in lag and 50% reduction in maximum OD_600_ as compared to wild type (Figure 3B). Vanillate is more toxic than ferulate to KT2440,^42^ such that this exacerbated vanillate accumulation may account for the increased toxicity of ferulate observed for Δ*oprF* (Figure 2A).

MV production was compared across all strains during growth on mixed HCAs (Figures 3C, S4, S5 and Excel file 1), and additionally wild-type MV production was compared with and without HCAs to evaluate the effect of HCAs on MV production (Figure S6 and Excel file 1). Wild-type KT2440 cultivations produced 1.4-fold more MVs during growth on HCAs compared to glucose only (Figure S6), which agreed with prior work that observed increased MVs during growth on corn stover APL^43^ and supported the hypothesis that aromatic compounds trigger vesiculation in KT2440. As observed on glucose alone, ΔPP_4669 maintained a hypovesiculation phenotype with 1.4-fold lower MV production as compared to KT2440 in the presence of HCAs (Figure 3C). For the hypervesiculating strains, Δ*oprF* had 1.6-fold higher particle counts relative to KT2440 (Figure 3C). Although the fold difference in particle counts in the presence of HCAs was not significant in the case of Δ*oprI* relative to KT2440, Δ*oprF* induced greater vesicle formation across both media compositions.

On both media compositions, Δ*oprI* MVs consistently had a particle size distribution shifted toward larger MVs whereas both Δ*oprF* and ΔPP_4669 had MVs with diameters shifted toward smaller MVs (Figure 3D). Although the mean size of the particle size distribution were only shifted 19–27 nm for the knockout strains relative to the mean size of 140 nm for KT2440, binning the MV sizes as demonstrated previously^58^ emphasizes that Δ*oprI* had 3-fold more MVs that were greater than 200 nm in diameter and Δ*oprF* and ΔPP_4669 had 2.4- and 2.1-fold more MVs in the 50–100 nm range (Figure S5). Thus, the deletion strains altered the formation of MVs as well as the extracellular dynamics of aromatic compound usage during growth on HCAs, leading to a need to understand whether the genetic manipulations also altered MV protein composition.

### Engineered Vesiculation Alters the Quantity and Composition of the MV Proteome

Manipulating MV formation through gene deletions raises the question of whether this genetic manipulation alters the MV protein cargo, which could have implications for the deployment of these strategies as genetic engineering tools. To evaluate this, we compared the cellular and MV proteome across the deletion strains. Notably, the vesicle-free secretome was not analyzed, but protein secretion separate from MV-mediated routes may be of future interest in the context of applying these tools. The strains were grown on glucose and HCAs, as described above, until midexponential phase, centrifuged, and fractionated into cell pellet and supernatant. MVs were harvested from the clarified supernatant as described above, and the proteomes of the cell and MV fractions were analyzed (Figure 4A).

**Figure 4:**
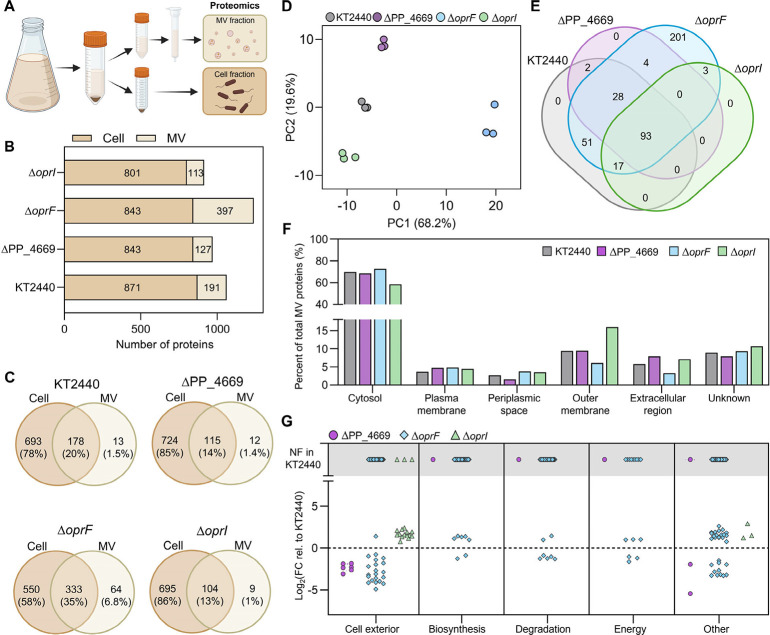
Differences in protein packaging into MVs between deletion strains and wild-type KT2440. (A) Schematic representation of the preparation of the cell and MV fractions for proteomics analysis. Schematic was generated using BioRender.com. (B) Total number of proteins measured in the cell and MV fractions. (C) Venn diagram of the number of proteins that were distinct or overlapped between the cell and MV fractions. (D) Principal component analysis of the MV fractions. (E) Venn diagram of the number of proteins that were distinct or overlapped between the MV factions. (F) Percentage of proteins classified in a specific cellular location relative to the total proteins in the MV fraction for each strain. (G) Statistically significant (*p* < 0.1) fold changes (FC) in protein abundance for the MV fractions for the deletion strains relative to KT2440 categorized by functionality. Proteins only found in deletion strains and not found (NF) in KT2440 are depicted in the gray portion. For F and G, the subsystems were obtained using MetaCyc classifications.^59^ All data are from three biological replicates. Proteomics measurements are provided in Excel file 1.

The total number of proteins measured in the cellular fraction was comparable between strains, ranging between 800 and 870 proteins (Figure 4B). However, in the MV fraction, Δ*oprF* had over 200 more detected proteins than KT2440 or the other knockout strains (Figure 4B). Over 93% of proteins in the MV fraction overlapped with the cellular fraction, with 1.0–6.8% of the total proteins being uniquely identified in the MV fraction (Figure 4C), supporting that the protein content in the MV fraction is representative of the proteins in the cell. Although the principal component analysis of the cellular proteome of the strains did not indicate clustering driven by genotype (Figure S7), the MV proteomes had close agreement between biological replicates and the data variation was driven strongly by genotype (Figure 4D). Accordingly, we further explored the differences in the protein composition in the MV fractions of the strains through presence/absence analysis as well as differential protein abundance.

Binary comparisons of the proteins identified in the MV fractions for KT2440 and the three deletion strains demonstrated that the protein composition of the MVs for wild-type KT2440, Δ*oprI*, and ΔPP_4669 were more closely associated with each other than to Δ*oprF* (Figure 4D,E). In particular, the alignment of protein quantity, composition, and differential abundance for KT2440 and ΔPP_4669 indicates that deletion of PP_4669 did not substantially alter the MV protein packaging (Figure 4D–G). Alternatively, 201 unique proteins were identified for the MV fraction of Δ*oprF* (Figure 4E), of which 76% were cytosolic proteins (Figure S8). This indicates that Δ*oprF* packaged a greater variety of cytosolic proteins into the MV fraction than KT2440 (289 versus 133 identified cytosolic proteins, respectively), despite only a 5% proportional increase in cytosolic proteins in the MV fraction relative to KT2440 (Figure 4F). Proteins across functional classifications of biosynthesis, degradation, and energy were all differentially abundant in the MV fraction of Δ*oprF* as compared to wild-type cultivations (Figure 4G), indicating no strong enrichment of a functional category. Thus, while MVs from Δ*oprF* cultivations contained 206 more proteins than wild-type MVs, no functional enrichment was apparent, suggesting increased but not differential protein cargo packaging was triggered by Δ*oprF*.

In contrast, MVs from Δ*oprI* cultivations had only three unique proteins compared to KT2440 MVs (Figure 4E) but the MV proteome was enriched in outer membrane proteins (1.7-fold greater than KT2440 and 2.7-fold greater than Δ*oprF*, Figure 4F). Cell exterior proteins (Biocyc subsystem including transport proteins, outer membrane proteins, periplasmic proteins, flagellar proteins, etc.) were 2.5- to 5-fold higher in abundance in MVs from Δ*oprI* relative to KT2440 cultivations (Figures 4G and S9). Conversely, Δ*oprF* had 1.5- to 32-fold lower cell exterior protein abundance relative to KT2440 (Figure 4G).

Lastly, although there were slight differences in the aromatic compound utilization for Δ*oprF* and Δ*oprI* compared to KT2440 (Figure 3B), there were no significant differences in the aromatic catabolic pathway proteins in the cell fractions of the strains (Excel file 1). In the Δ*oprF* MVs, some aromatic catabolic proteins (namely, PcaH and PcaG) had up to 2.4-fold decreased abundance compared to KT2440 whereas others (VanA, VanB, PcaC, and PcaD) were only found in Δ*oprF* MVs (Figure 3A and Excel file 1). For Δ*oprI*, there were no appreciable differences in aromatic compound pathway enzymes in the MV fraction compared to KT2440 (Excel file 1). Thus, the differences in utilization of HCAs between strains do not appear to be due to differential protein abundances in the cell or MV fractions.

Overall, these data show that Δ*oprF* increased the quantity of proteins in the MV fraction including cytosolic proteins across diverse Gene Ontology classifications whereas Δ*oprI* produced MVs with a similar amount of proteins to wild-type MVs but with outer membrane protein enrichment (Figure 4E–G). The high amount of cytosolic protein packaging in Δ*oprF-*derived MVs may be beneficial for nontargeted secretion of highly expressed cytosolic proteins, facilitating applications such as extracellular protein recovery without the use of tags.

### Hypervesiculation Improves Protein Export with Tag-Specific Effects

Protein secretion mechanisms in KT2440 are not well established,^60^ such that genetic tools to manipulate MV formation and protein targeting to MVs offer opportunities to act as tools for protein export applications. Previously, genetic engineering of hypervesiculation in and has been utilized to improve small molecule secretion, such as for the natural products such as β-carotene and phenazine-1-carboxylic acid.^8,9^ Hypervesiculation phenotypes in were also associated with higher extracellular green fluorescent protein signal,^11^ implying that engineered vesiculation can be used not only for small molecule secretion but also as a method for protein export. However, MV production as a protein secretion strategy for KT2440 has not been similarly explored. Notably, the spatiotemporal fate of MV-secreted proteins due to the potential for MV lysis or trafficking remains an open question and requires super high-resolution imaging to address. Thus, here we measured the effect of genetic strategies on the total secreted protein contained in the extracellular milieu as a fraction of total intra- and extra-cellular signal.

We first assessed nontargeted protein secretion via hypervesiculation by measuring fluorescence from a mNeonGreen (mNG) reporter overexpressed on pBTL-2 (Table S3). The mNG fluorescence signal was measured in the washed and resuspended cell pellet and the clarified supernatant (Figure S10). The fraction of extracellular signal relative to the total measured signal was compared between the hypervesiculation strains and KT2440 (Figure 5). The extracellular fraction of the total mNG signal significantly increased by 2.9- and 2.0-fold for Δ*oprF* and Δ*oprI*, respectively, highlighting that both gene deletions alone can be used to export highly expressed proteins from the cytosol to the extracellular milieu.

**Figure 5:**
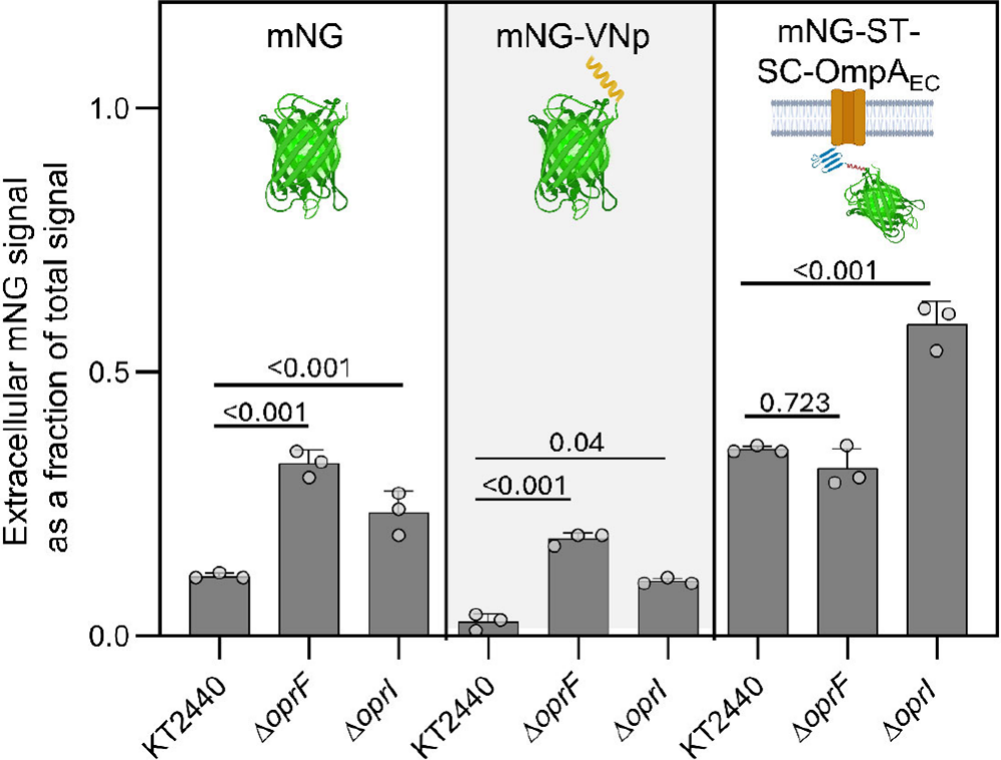
Protein secretion in wild-type KT2440, Δ*oprF*, or Δ*oprI* with untagged, VNp-tagged, and SC-ST-tagged mNeonGreen (mNG). mNG was measured in the extracellular fraction relative to the total cumulative signal of the clarified supernatant and cell. Individual points are shown for each of the biological triplicates; error bars represent standard deviation across all measurements. Statistically significant differences (*p* < 0.05) were determined by one-way ANOVA followed by Tukey HSD post hoc tests. All raw data can be found in Excel file 1.

Next, we explored targeted protein secretion into the extracellular milieu through a combination of hypervesiculation and protein tags. Previous work in utilized vesicle nucleating peptide (VNp) tags or SpyCatcher-SpyTag (SC-ST) bioconjugation systems with the OmpA outer membrane porin as an anchor.^12,28^ Here, we implemented and compared the two systems by (1) fusing VNp to mNG or (2) fusing OmpA from to SC paired with mNG fused to ST. Although the VNp tag decreased the fractional extracellular signal 3.6-fold relative to the unmodified mNG (*p* = 0.016), the Δ*oprF* and Δ*oprI* strains displayed 6.0- and 3.3-fold increases, respectively, in mNG-VNp fractional extracellular signal compared to KT2440 (Figure 5), supporting that hypervesiculation improves targeted protein export. Whether the previously reported truncated VNp tags^28^ are more effective than the whole VNp tag for protein export in KT2440 remains to be elucidated.

In contrast to the VNp tag, addition of the SC-ST system into KT2440 increased the extracellular signal for mNG 3.1-fold compared to unmodified mNG (*p* < 0.001) (Figure 5). Notably, Δ*oprI* paired with the SC-ST system increased export 1.7-fold compared to the SC-ST system in KT2440 and 5.4-fold compared to mNG alone (Figure 5). Conversely, Δ*oprF* did not improve the export in the SC-ST background. The OmpA protein from expressed in the SC-ST system is an outer membrane porin with 34% identity to OprF (Table S2), and thus the expression of OmpA may counteract the vesiculation effects from the deletion of *oprF*. In contrast, Δ*oprI* MVs were enriched in outer membrane proteins compared to wild-type KT2440 (Figure 4F,G), and thus packaging of OmpA into Δ*oprI* MVs may be similarly enriched. Ultimately, these data demonstrate that hypervesiculation can be used to improve protein export in KT2440 with nontargeted, VNp-targeted, and SC-ST targeted systems, and that the hypervesiculation strategy should be carefully paired with protein tags for maximal secretion.

### Overexpression of Membrane Biosynthesis Genes Improved Membrane Permeability in Δ*oprF*

Lastly, we considered whether the high membrane permeability and the poor cellular growth observed in Δ*oprF* could be mitigated by upregulating membrane biosynthesis genes as cellular membrane loss could be substantial in hypervesiculating strains. Membrane biosynthesis proteins were not increased in Δ*oprF* relative to KT2440 (Figure 6A,B). Instead, we found that the glycerol-3-phosphate dehydrogenase GpsA, which reduces dihydroxyacetone phosphate to glycerol-3-phosphate as an initial step in the glycerophospholipid biosynthesis pathway, was 2.0-fold lower in Δ*oprF* compared to KT2440, suggesting that a downregulation of this pathway may contribute to membrane permeability in this strain (Figure 6A,B). Thus, to determine how to improve strain fitness while maintaining vesiculation, we overexpressed *gpsA* along with three other enzymes relevant to membrane biosynthesis – *plsBC*, *cdsA*, or *accABCD* (Figure 6A) – in the Δ*oprF* background and measured the effect on membrane permeability and MV secretion.

**Figure 6:**
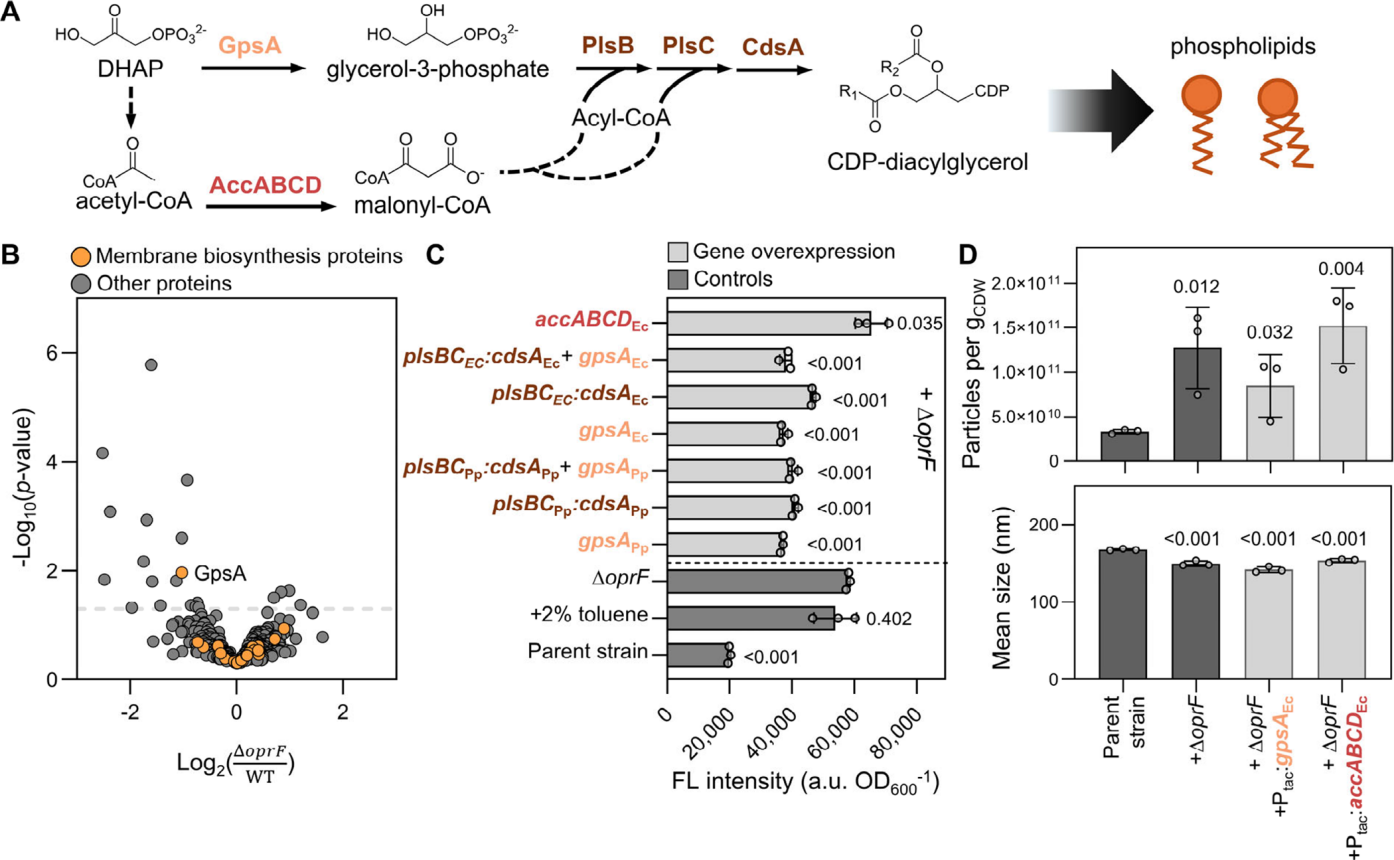
Identification and overexpression of membrane biosynthesis genes that improve or exacerbate membrane permeability. (A) Schematic of proteins targeted in membrane biosynthesis pathways for overexpression. (B) Volcano plot of differentially abundant proteins in the cellular fractions of Δ*oprF* relative to KT2440. Proteins belonging to membrane biosynthesis pathways are highlighted in orange. (C) The propidium iodide assay was used to assess the membrane permeability of the overexpression strains illustrated in light gray. The controls, depicted in dark gray, included the parent strain (AG5577),^62,63^ which acted as a reference for intact membranes, and Δ*oprF* and the parent strain exposed to 2% toluene, which were references for permeabilized membranes. Individual points are shown for each of the biological triplicates; error bars represent standard deviation across all measurements. Statistically significant differences (*p* < 0.05) were determined by one-way ANOVA followed by Dunnett’s multiple comparisons test with Δ*oprF* as the control. (D) Particle counts and size distributions measured by NTA for the control strains in dark gray and the overexpression targets in light gray. Individual data points are illustrated for the three biological replicates; error bars depict the standard deviation. Significant differences (*p* < 0.05) were determined with an unpaired one-tailed *t*-test to test the hypothesis that overexpression of membrane biosynthesis genes in Δ*oprF* produce a greater number of MVs than the parent strain. All raw data are provided in Excel file 1. Abbreviations used in A: DHAP, dihydroxyacetone phosphate; CDP, cytidine diphosphate.

Due to potential benefits of exogenous proteins averting native regulation,^61^ GpsA or PlsBC:CdsA enzyme homologues from were compared to the native KT2440 enzymes (Figure 6C). Whether the native gene or in origin, membrane permeability was significantly decreased (*p* < 0.001), relative to Δ*oprF* alone, by 37% when overexpressing *gpsA*, 20–30% when overexpressing *plsBC:cdsA,* or 31–35% when overexpressing the combination of *gpsA* and *plsBC:cdsA* (Figure 6C). Notably, overexpressing *gpsA* with *plsBC:cdsA* did not improve the permeability more than *gpsA* alone (Figure 6C). Although the overexpression of genes in the glycerophospholipid biosynthesis pathway improved the membrane integrity compared to Δ*oprF*, the membrane permeability of these strains was still around 2-fold higher (*p* < 0.001) than the parent strain (Figure 6C and Excel file 1), indicating improvements could still be made to enhance the integrity of the membrane in these strains. Additionally, *accABCD* from was overexpressed in Δ*oprF* to examine the impact of targeting the initiation of the fatty acid de novo biosynthesis pathway instead of initial enzymes in the glycerophospholipid biosynthesis pathway. In contrast to overexpressing *gpsA*, *accABCD* overexpression increased the membrane permeability by 13 and 228% relative to Δ*oprF* and the parent strain, respectively (Figure 6C). Previously, β-carotene export in was improved when *accABCD* was overexpressed in hypervesiculating production strains,^8^ implying the potential association of *accABCD* overexpression with increased vesiculation relative to a deletion strain alone.

To assess whether the *gpsA* and *accABCD* overexpression in Δ*oprF* maintained an increase in vesiculation compared to the parent strain, the strains were grown to an OD_600_ of 1.0–1.9 on 20 mM glucose before MVs were extracted from the clarified supernatant. Relative to the parent strain, Δ*oprF,* Δ*oprF* plus *gpsA*_*EC*_ overexpression, and Δ*oprF* plus *accABCD*_*EC*_ overexpression had, respectively, 3.8-, 2.6-, and 4.6-fold greater particle counts (Figure 6D). Although overexpression of *gpsA*_*EC*_ improved the membrane permeability (Figure 6C), this strain did not significantly alter the vesicle counts relative Δ*oprF* alone (unpaired two-tailed *t*-test; *p* = 0.272). Additionally, the distribution and mean size of all the engineered strains were consistently smaller than the parent strain as demonstrated earlier for Δ*oprF*, suggesting that the mechanism for blebbing remained consistent with the overexpression of the membrane biosynthesis genes (Figures 3D and 6D). Even though the 13% increased membrane permeability of the *accABCD* overexpression strain was paired with no change in the particle counts relative to Δ*oprF* (unpaired two-tailed *t*-test; *p* = 0.528), the overexpression of *accABCD* may have a more substantial impact on MV functionality as was seen here for protein export by deleting *oprI* and previously for β-carotene export by overexpressing *accABCD* in .^8^ Thus, additional manipulations of the fatty acid biosynthetic pathway may be investigated to enhance vesiculation further. Overall, selective targeting of membrane biosynthesis genes can be employed as a strategy to reduce membrane instability in hypervesiculating strains.

## Conclusions

In summary, we found that the deletion of *oprF* encoding an outer membrane porin or *oprI* encoding a lipoprotein stimulated the production of MVs in KT2440, and had distinct impacts on cellular growth, membrane integrity, MV size distributions, and MV protein composition. Namely, deletion of *oprF* increased the quantity of MVs, increased the packaging of proteins into the MVs, weakened the membrane integrity, and diminished the growth on and tolerance to aromatic compounds as compared to wild-type KT2440. Alternatively, deletion of *oprI* maintained cellular growth, catabolic, and membrane phenotypes while also generating a moderate hypervesiculation phenotype with vesicles generally larger in size and enriched in outer membrane proteins. Both hypervesiculation genetic targets were shown to increase the secretion of proteins, although the SC-ST bioconjugation system using OmpA_EC_ was more effective when paired with Δ*oprI*. Finally, overexpression of the glycerol-3-phosphate dehydrogenase *gpsA* reduced the membrane permeability of Δ*oprF* and represents a strategy to mitigate the impact of hypervesiculation on strain performance. These strategy-specific outcomes have opportunities for diverse applications from enzyme production, cell-free bioprocesses, and vaccine development, where strain fitness is less important than MV production, to polymer degradation or small hydrophobic molecule production, where a balance between strain fitness and MV production is required. Altogether, this study provides a framework for pairing genetic engineering strategies in KT2440 to maximize MV and protein secretion and minimize negative effects on cellular phenotypes, representing promising new tools for biotechnological applications.

## Materials and Methods

### Bacterial Strains and Media

The strains used in this study included KT2440 (ATCC 47054) and genetically engineered derivatives of this strain (Table S3). Transposon mutants of KT2440 (Putida_ML5) were extracted from an arrayed RB-TnSeq library generated using the plasmid pKMW3 as described previously.^49,50,64^ Gene disruptions were verified by Sanger sequencing the associated molecular barcodes at GENEWIZ (Azenta USA). All strains were stored in 25% v/v glycerol at −80 °C.

Strains were revived by directly inoculating frozen stocks into lysogeny broth (LB) medium (Lennox) at 30 °C. Cells were cultivated in either LB medium or a modified M9 minimal media (6.78 g/L Na_2_HPO_4_ 3 g/L KH_2_PO_4_, 0.5 g/L NaCl, 1 g/L NH_4_Cl, 2 mM MgSO_4_, 100 μM CaCl_2_, and 18 μM FeSO_4_). Glucose was supplemented, as described for each experiment, into the M9 minimal media from a filtered 2 M solution in water to a final concentration of 20 mM. All media with *p*-coumarate and ferulate were titrated with 5 M NaOH to solubilize and neutralize to a final pH of 7.0 before utilizing for cell cultures. For aromatic compound tolerance experiments, 200 mM of *p*-coumarate or ferulate was made in 20 mM glucose M9 minimal media and diluted to the tested aromatic compound concentrations (200,125, 75, 25, 0 mM). For shake flask experiments, a stock solution of 25 mM *p-*coumarate and ferulate was solubilized in M9 salts (6.78 g/L Na_2_HPO_4_ 3 g/L KH_2_PO_4_, 0.5 g/L NaCl, 1 g/L NH_4_Cl) before mixing with the other components to make a final concentration of 20 mM glucose, 12.5 mM *p-*coumarate, and 12.5 mM ferulate in M9 minimal media. *Pseudomonas* quinolone signal (2-heptyl-3-hydroxy-4­(1*H*)-*quinolone;* PQS) was prepared in methanol at a stock concentration of 5 mM. For experimental conditions containing PQS, the stock was spiked into individual flasks to a final concentration of 50 μM and the methanol was evaporated in each flask under sterile conditions overnight before addition of experimental minimal media. All media was filter sterilized (0.2 μm pore size) before use. All chemicals were purchased from Sigma-Aldrich.

### Plasmid and Strain Construction

Construction details including plasmids, oligonucleotides, and strains are detailed in Tables S3–S5. All strains are derivatives of KT2440 (ATCC 47054). In brief, markerless gene deletions were performed using the nonreplicative pK18sB vector with 1000 base pair homology regions.^65^ Overexpression of mNG alone or paired with a MV targeting system was conducted using pBTL-2 plasmids.^66^ For genome integration to overexpress genes involved in membrane biosynthesis with the constitutive *tac* promoter, either pJH0419 or pJH0210 were utilized to integrate genes in the parent strain AG5577 containing a total of nine unique *attB* sites.^62,63^ For all overexpression plasmid constructs, ribosome binding sites were optimized for each gene sequence as described previously.^67^ All plasmids were synthesized by TWIST Biosciences, except pTM018, pTM007, and pTM008 that were assembled using the New England Biolabs Hifi Gibson Mix (Table S5).

Competent cells were prepared as described previously,^68^ and electroporated with the 100 to 500 ng of plasmid DNA. Cells were recovered for 1–2 h in sterile SOC Outgrowth Medium (New England Biolabs) at 30 °C. Markerless gene deletion was accomplished by the *sacB*/KanR counterselection as reported previously.^69^ Maintenance of the pBTL-2 plasmids in all growth conditions was achieved with 50 mg/L of kanamycin. All integrations of pJH0419 and pJH0210 vectors were achieved by serine recombinase-assisted genome engineering (SAGE) using the BxB1 or TG1 recombinase, respectively, and their corresponding antibiotic resistance cassette as established previously (Table S5).^62,63^ All SAGE integrations were conducted with the addition of 34 mg/L chloramphenicol. Double mutants were selected on 100 mg/L streptomycin, 50 mg/L kanamycin, and 34 mg/L chloramphenicol. Correct transformants were screened with colony polymerase chain reaction (cPCR) and confirmed with Sanger sequencing at GENEWIZ (Azenta USA) or Oxford Nanopore sequencing at Plasmidsaurus as described in Tables S3 and S4. Individual transposon mutants were extracted from a previously described arrayed randomly barcoded transposon mutant library in KT2440 (Putida_ML5).^49,50,64^ Each mutant culture was grown overnight at 30 °C in LB plus 50 mg/L of kanamycin, screened with PCR, and sanger sequenced to verify the barcode sequence of each mutant. Correct colonies for all strains were grown overnight at 30 °C in LB and stored as 20% (v/v) glycerol stocks at −80 °C.

### Bacterial Growth

For all experiments, seed cultures from glycerol stocks were revived in LB medium for 15–16 h, pelleted at 5000*g* for 5 min, washed in M9 salts, pelleted again, and resuspended in the experimental M9 minimal media at an optical density (OD_600_) of ∼0.1. All seed and experimental cultures were grown at 30 °C. For aromatic compound tolerance experiments, cells were grown in 200 μL in Corning 96-well plates for 30 h using the LogPhase600 instrument with 500 rpm shaking frequency and OD_600_ measurements every 20 min. For membrane permeability and mNG assays, cells were grown in 5 mL of culture medium in tubes with shaking at 220 rpm. For consumption profiling and MV extractions, cells were grown in unbaffled shake flasks (either 150 mL or 250 mL) with sponge caps at one-fifth total flask volume with shaking at 220 rpm. Aliquots of cell suspensions were collected for monitoring growth at OD_600_ and quantifying extracellular metabolites. Samples for metabolite quantification were collected by centrifuging for 5 min at >10,000*g* and filtering the supernatant through 0.22 μm nylon Costar Spin-X centrifuge tube filters (Corning). All extracellular metabolite samples were stored at −20 °C until analysis.

### MV Extraction and Quantification

Cells of KT2440 and derivative strains were cultivated in M9 minimal media with glucose alone or glucose plus HCAs until mid-to-late exponential phase. An enrichment of MVs was extracted from cell cultures as described previously.^43^ In brief, aliquots (25–30 mL) of cell culture were collected in sterile preweighed 50 mL centrifuge tubes and pelleted at 8000*g* for 20 min at 4 °C. The cell pellet for each strain from this first centrifugation step was stored at −80 °C until completely frozen, lyophilized for 24 h, and weighed to obtain the g_CDW_/mL used to normalize MV counts. The supernatants, from this first centrifugation step, were collected and transferred to new sterile 50 mL centrifuge tubes and spun again at 8000*g* for 20 min at 4 °C. The resulting supernatant was filtered through a 0.2 μm filter unit (ThermoFisher) to produce a cell-free clarified supernatant used for MV extractions. Enrichment of MVs from the clarified supernatant was conducted using the ExoBacteria MV Isolation Kit (System Biosciences). The enriched MV fraction, eluted in 1.5 mL, was analyzed directly or stored at −80 °C before quantification with nanoparticle tracking analysis (NTA). A maximum of one freeze–thaw cycle was conducted before analysis to minimize MV lysis. Control strains were always conducted in parallel with each experimental strain extraction.

For quantification of vesicle counts, the enriched MVs were 1:20 or 1:50 diluted in 0.22 μm-filtered phosphate buffered saline (PBS) to reach a particle concentration between 10^7^ and 10^9^ particles/mL. The samples were injected through a flow cell at a rate of 30 μL/min and analyzed on a NanoSight NS300 system (Malvern Panalytical, UK) equipped with a 638 nm laser with a 650 nm long-pass filter in the Analytical bioNanoTechnology Equipment Core Facility of the Simpson Querrey Institute for BioNanotechnology at Northwestern University. Each biological replicate was measured in three technical replicates. Data processing was performed on the Nanosight software (NTA 3.4.4). To obtain the particle counts per g_CDW,_ particle counts per mL were corrected by the concentration factor (25 or 30 mL eluted in 1.5 mL) before normalization to the g_CDW_ per mL that was measured for each replicate.

### Membrane Permeability Assessment

Cells were grown until midexponential in glucose only or glucose plus the HCAs. The OD_600_ of each culture was measured and recorded for normalization. To create a positive control for permeabilized cells, an aliquot of KT2440 at midexponential was incubated with 2% toluene for 30 min before pelleting and washing twice with PBS. All cell suspensions were pelleted at 5000*g* for 5 min and resuspended in PBS at a 2× concentration. A propidium iodide assay was conducted, as described previously,^57^ by incubating 500 μL of the cell suspensions with 5 μL of propidium iodide solution (0.1 mg/mL in miliQ H_2_O) for 10 min at room temperature. A 100 μL aliquot of each reacted cell suspension was transferred into a 96-well plate in technical duplicate. The fluorescence of DNA bound propidium iodide was measured on the Tecan microplate reader (Infinite 200Pro, Tecan Group Ltd.) at an excitation of 535 nm and an emission of 617 nm with multiple reads per well (total of eight reads).

### Quantification of Glucose and Aromatic Compounds

Quantification of glucose and aromatic acids (*p*-coumaric acid, ferulic acid, 4-hydroxybenzoic acid, vanillic acid, 4-hydroxybenzaldehyde, vanillin, and protocatechuic acid) were analyzed, respectively, as described previously.^70,71^ In brief, glucose was analyzed using an Agilent 1200 Series system performing high performance liquid chromatography with refractive index detection (HPLC-RID). Isocratic separation was conducted at a flow rate of 0.6 mL/min with a Bio-Rad Aminex HPX-87H Ion Exclusion Column (300 × 8.7 mm, 9 μm particle size) maintained at 55 °C. For aromatic acids, reverse phase chromatographic separation was conducted on an Agilent 1290 series ultrahigh performance liquid chromatography system combined with a diode array detector (UHPLC-DAD). A Phenomenex Kinetex reverse phase analytical column (2.1 × 100 mm; 1.7 μm particle size) was utilized with a flow rate of 0.8 mL/min and the temperature maintained at 35 °C. Linear calibration curves for each analyte of interest had an *r* ^2^ coefficient ≥ 0.995 and were used to quantify glucose and aromatic acids in the extracellular medium.

### Proteomics Analysis

For cellular proteomics analyses, proteins were extracted from pelleted whole-cell biomass by vortexing in a lysis buffer containing SDS (1%), Tris (200 mM, pH 8.0) and dithiothreitol (DTT; 10 mM) for 20 min at 95 °C, followed by alkylation of cysteine thiols in 40 mM iodoacetamide. For MV samples, cell-free supernatants were first concentrated onto spin filters (Sartorius Vivafree 500, 30 kDa nominal cutoff), then solubilized in lysis buffer, heated and reduced/alkylated as above. Vesicle protein extracts were then precipitated in glass centrifuge tubes with 4 volumes of cold acetone (−20 °C, overnight), centrifuged (7197 *g*, 1 h), and protein pellets redissolved in 8 M urea/0.2% deoxycholate/1 M ammonium bicarbonate.

Protein extracts were purified and digested using a modified enhanced filter-aided sample preparation protocol^72^ using Sartorius Vivafree 500 concentrators (30 kDa cutoff). The purified proteins were then digested with MS-grade trypsin at 37 °C overnight. Peptides eluted from the concentrator were dried in a vacuum concentrator and isotopically labeled at both C and N termini using the diDO-IPTL method for downstream quantitative analysis. In brief, C termini were labeled with either ^16^O or ^18^O, while the N termini were labeled with either un- or dideuterated formaldehyde (CH_2_O or CD_2_O).^73^ An internal standard was prepared from pooled peptide aliquots labeled with CH_2_O/^18^O. ^16^O-labeled peptide samples were mixed 1:1 (v/v) with ^18^O-labeled internal standard for quantification.

For LC-MS analysis, peptides were separated through a monolithic capillary C_18_ column (GL Sciences Monocap Ultra, 100 μm inside diameter × 200 cm length) using a water–acetonitrile and 0.1% formic acid gradient (2–50% acetonitrile over 180 min) at a flow rate of 360 nL/min on a Dionex Ultimate 3000 LC system with nanoelectrospray ionization (Proxeon Nanospray Flex). High resolution mass spectra of MS1 parental ion full scan (120,000 *m*/Δ*m*) with fragment ion scans of selected precursors were collected on an Orbitrap Elite mass spectrometer (Thermo Fisher) operating in a data-dependent acquisition (DDA) mode. The analysis of mass spectra was performed using MorpheusFromAnotherPlace (MFAP)^73^ with the annotated predicted proteome of KT2440. The mass tolerance of precursor and product ions on MFAP was set at 20 ppm and 0.6 Da, respectively. Modifications were made to include static cysteine carbamidomethylation and variable methionine oxidation, N-terminal (d4)-dimethylation and C-terminal ^18^O_2_. The false discovery rate for peptide spectrum matches was controlled to <0.5% using target-decoy searching. The relative abundance and standard errors of proteins were calculated in R using the Arm postprocessing scripts for diDO-IPTL data (github.com/waldbauerlab).

### mNeonGreen Measurements

Cells were grown in LB for 24 h in tubes before collection of the cell suspension, measurement of the OD_600_ of the cell suspension for normalization, and centrifugation at 5000*g* for 5 min of a 1 mL aliquot to separate the cell and supernatant. The cell pellet was additionally washed once in M9 media (no carbon source) before resuspension in M9 media to an equal volume as the initial cell suspension. The supernatant was filtered through 0.22 μm nylon Costar Spin-X centrifuge tube filters (Corning) to ensure the removal of cells. A 200 μL aliquot of each filtered supernatant and resuspended cell pellet was loaded onto a 96-well plate in biological triplicate. The mNG fluorescence signal was measured at an excitation of 495 nm and an emission of 525 nm with multiple reads per well on the Tecan microplate reader (Infinite 200Pro, Tecan Group Ltd.). The mNG signal was first normalized by the OD_600_ and then the background signal noise from the empty vector pBTL-2 controls, prepared the same way to separate cell and extracellular fractions, was subtracted out. Finally, to determine if there were differences in protein export between strains, the normalized extracellular signal was divided by the summed signal in the normalized extracellular fraction and the cell fraction.

## Supplementary Material





## Data Availability

Experimental data are presented in the manuscript or provided in the Supporting Information and Excel file 1. Proteomics data was deposited in the MassIVE repository (data set MSV000097014; password for review: KT2440omv) and proteomeXchange (data set PXD060399).

## Notes

Raw data for individual replicates corresponding to the figures including nanoparticle analysis, extracellular metabolite depletion profiles, proteomics, membrane permeability assay, and mNeonGreen assay (XLS)
Table S1, putative gene knockout targets in KT2440 to enhance vesiculation; Table S2, protein sequence identity of OmpA from K12 to KT2440 genes; Table S3, strains utilized in this study and corresponding construction details; Table S4, oligonucleotides utilized in this study; Table S5, plasmids utilized in this study; Table S6, sequences for mNeonGreen, tags, and codon-optimized genes; Figure S1, particle count per g_CDW_ for KT2440 and knockout strains corresponding to data presented in Figure 1B; Figure S2, OD_600_ measurements of extracted MVs from KT2440 and knockout strains; Figure S3, particle count per gCDW for WT, ΔPP_4669, and ΔPP_1502; Figure S4, particle count per gCDW for KT2440 and knockout strains corresponding to data presented in Figure 3C; Figure S5, sizes of MVs corresponding to particle counts in Figure S4; Figure S6, particle count per gCDW for KT2440 grown on 20 mM glucose alone or 20 mM glucose plus 12.5 mM p-coumarate and 12.5 mM ferulate; Figure S7 and Figure S8, principal component analysis of the cellular fractions; Figure S9, heatmap of outer membrane proteins with differential abundance; and Figure S10, mNeonGreen (mNG) fluorescence signal for the cellular fraction and the extracellular fraction (PDF)

## Abbreviations

APL: alkaline pretreated liquor
g_CDW_: gram cell dry weight
HCAs: hydroxycinnamic acids
KT2440: KT2440
mNG: mNeonGreen
MV: membrane vesicle
NTA: nanoparticle tracking analysis
PQS: *Pseudomonas* quinolone signal
SC: SpyCatcher
ST: SpyTag
Vnp: vesicle nucleating peptide
