# Supplementary material for: Engineered Membrane Vesicle Production via oprF or oprI Deletion Has Distinct Phenotypic Effects in Pseudomonas putida
Source: ACS Synth Biol. 2025 Jul 3;14(7):2739–52. doi: 10.1021/acssynbio.5c00171 (PMC12281618; doi:10.1021/acssynbio.5c00171)
Supplement: Supplementary file 2 [file sb5c00171_si_002.pdf]

Engineered membrane vesicle production via *oprF* or *oprI* deletion has distinct phenotypic effects in *Pseudomonas putida*

Rebecca A. Wilkes[1], Tarryn E. Miller[1], Jake Waldbauer,[2] Nanqing Zhou[3], Beth DiBiase[3], Neha P. Kamat[3], Ludmilla Aristilde[3], Gregg T. Beckham[1], and Allison Z. Werner[1*]

(1) Renewable Resources and Enabling Sciences Center, National Renewable Energy Laboratory, Golden, CO, USA, (2) Department of the Geophysical Sciences, University of Chicago, Chicago, IL USA, (3) Department of Civil and Environmental Engineering, McCormick School of Engineering and Applied Science, Northwestern University, Evanston, IL USA
*To whom correspondence should be addressed: allison.werner@nrel.gov

Contents

Table S1. Putative gene knockout targets in *P. putida* KT2440 to enhance vesiculation. 2

Table S2. Protein sequence identity of OmpA from *E. coli* K12 to *P. putida* KT2440 genes 3

Table S3. Strains utilized in this study and corresponding construction details. 4

Table S4. Oligonucleotides utilized in this study. 7

Table S5. Plasmids utilized in this study. 9

Table S6. Sequences for mNeonGreen, tags, and codon-optimized genes 13

Figure S1. Particle count per g_{CDW} for KT2440 and knockout strains corresponding to data presented in Fig. 1B. 21

Figure S2. OD_{600} measurements of extracted MVs from KT2440 and knockout strains. 22

Figure S3. Particle count per gCDW for WT, ΔPP_4669, and ΔPP_1502 23

Figure S4. Particle count per gCDW for KT2440 and knockout strains corresponding to data presented in Fig. 3C. 24

Figure S5. Sizes of MVs corresponding to particle counts in Fig. S3 were binned into three size ranges and presented as a percentage of the total MV population. 25

Figure S6. Particle count per gCDW for KT2440 grown on 20 mM glucose alone or 20 mM glucose plus 12.5 mM p-coumarate and 12.5 mM ferulate 26

Figure S7. Principal component analysis of the cellular fractions 27

Figure S8. The percentage of proteins classified in a specific cellular location relative to the total number (201) of unique proteins found in the MV fraction for Δ*oprF* 28

Figure S9. Heatmap of outer membrane proteins in MV fraction with differential abundance 29

Figure S10. mNeonGreen (mNG) fluorescence signal for the cellular fraction and the extracellular fraction normalized by the OD600 of the cell suspension 30

Table S1. Putative gene knockout targets in *P. putida* KT2440 to enhance vesiculation.

Candidate protein target	Locus tag/s in *P. putida* KT2440	Cellular location (GO term)	Proposed mechanism	Precedence	Reference
TolA	PP_1221	Outer membrane	Deletion of *tolA* will disrupt the Tol cell envelope complex causing the destabilization of the membrane by disrupting the outer membrane to peptidoglycan to inner membrane linkage.	*Salmonella typhimurium*	([1])
				Escherichia coli	([2,3])
TolR	PP_1220	Plasma membrane	Deletion of *tolR* will disrupt the Tol cell envelope complex causing the destabilization of the membrane by disrupting the outer membrane to peptidoglycan to inner membrane linkage.	*Escherichia coli*	([2])
				Salmonella enterica serovar Typhi	([4])
TolB	PP_1222	Periplasmic space	Deletion of *tolB* will disrupt the Tol cell envelope complex causing the destabilization of the membrane by disrupting the outer membrane to peptidoglycan to inner membrane linkage.	*Salmonella typhimurium*	([1])
				Escherichia coli	([3])
OprL (Pal)	PP_1223	Outer membrane	Deletion of *oprL* will destabilize the membrane by disrupting the outer membrane to peptidoglycan linkages.	*Salmonella typhimurium*	([1])
				Escherichia coli	([3])
OmpA	PP_1121	Outer membrane	Deletion of *ompA* will destabilize the outer membrane by disrupting the outer membrane tethering to the peptidoglycan layer.	*Acinetobacter baumannii*	([5])
	PP_4669				
	PP_1122				
	PP_1502			*Salmonella typhimurium*	([1])
	PP_4198				
OprF	PP_2089	Outer membrane	Deletion of *oprF* will destabilize the outer membrane by disrupting the outer membrane tethering to the peptidoglycan layer.	*Pseudomonas aeruginosa*	([6])
OprI	PP_2322	Outer membrane	Deletion of *oprI* will destabilize the outer membrane by disrupting the outer membrane tethering to the peptidoglycan layer.	*Pseudomonas aeruginosa*	([6])
				Pseudomonas putida	([7])
MlaA (VacJ)	PP_2163	Outer membrane	Deletion of *mlaA* will disrupt the Mla pathway causing phospholipid accumulation in the outer leaflet and increase the outer membrane curvature.	*Escherichia coli*	([8])
				Haemophilus influenzae<br>*Vibrio cholerae*	([9])
MlaD	PP_0960	Extracellular region	Deletion of *mlaD* will disrupt the Mla pathway causing phospholipid accumulation in the outer leaflet and increase the outer membrane curvature.	*Veillonella parvula*	([10])

Table S2. Protein sequence identity of OmpA from *E. coli* K12 to *P. putida* KT2440 genes

***E. coli* K12**		***P. putida* KT2440**			
**Protein reference**	**UniProt Accession No.**	**Gene name**	**Protein name**	**UniProt Accession No.**	**Identity (%)**
OmpA	P0A910	PP_1122	OmpA family protein	Q88NT2	42.1
		PP_1121	OmpA family protein	Q88NT3	38.5
		PP_1502	OmpA family protein	Q88MR7	38.9
		PP_4669	OmpA family protein	Q88DZ9	39.0
		PP_4198	OmpA family protein	Q88FA0	34.5
		oprF (PP_2089)	Outer membrane porin F	Q88L46	34.2

Table S3. Strains utilized in this study and corresponding construction details.

Strain	Genotype	Construction details	Reference
KT2440	Wild-type *Pseudomonas putida* KT2440	n/a	ATCC® 47054
RW29	*P. putida* KT2440 Δ*oprF*	pRW007 was transformed into KT2440. Deletion of *oprF* (PP_2089) was confirmed by colony PCR with oRW037 and oRW038 (Tm=68°C, 2.3 kB) followed by Sanger sequencing.	This study
RW30	*P. putida* KT2440 Δ*oprI*	pRW008 was transformed into KT2440. Deletion of *oprI* (PP_2322) was confirmed by colony PCR with oRW041 and oRW042 (Tm=67°C, 2.3 kB) followed by Sanger sequencing.	This study
RW25	*P. putida* KT2440 ΔPP_1121	pRW005 was transformed into KT2440. Deletion of PP_1121 was confirmed by colony PCR with oRW029 and oRW030 (Tm=61°C, 2.3 kB) followed by Sanger sequencing.	This study
RW27	*P. putida* KT2440 ΔPP_4669	pRW006 was transformed into KT2440. Deletion of PP_4669 was confirmed by colony PCR with oRW033 and oRW034 (Tm=63°C, 2.5 kB) followed by Sanger sequencing.	This study
RW95	*P. putida* KT2440 ΔPP_1502	pRW039 was transformed into KT2440. Deletion of PP_1502 was confirmed by colony PCR with oRW127 and oRW128 (Tm=67°C, 2.5 kB) followed by Sanger sequencing.	This study
RW02	*P. putida* KT2440 PP_1122::$Tc1_{1285068}$(Km)[b]	Strain was obtained directly from the arrayed transposon mutant library. The barcode was PCR amplified with Barseq_P1 and Barseq_P2 (Tm=55°C, <0.2 kB) and sequence confirmed with BarSeq_SangerSeq.	([11])
RW03	*P. putida* KT2440 PP_1502::$Tc1_{1707548}$(Km)[b]	Strain was obtained directly from the arrayed transposon mutant library. The barcode was PCR amplified with Barseq_P1 and Barseq_P2 (Tm=55°C, <0.2 kB) and sequence confirmed with BarSeq_SangerSeq.	([11])
RW04	*P. putida* KT2440 PP_4198::$Tc1_{4744244}$(Km)[b]	Strain was obtained directly from the arrayed transposon mutant library. The barcode was PCR amplified with Barseq_P1 and Barseq_P2 (Tm=55°C, <0.2 kB) and sequence confirmed with BarSeq_SangerSeq.	([11])
RW05	*P. putida* KT2440 *mlaA*::$Tc1_{2469635}$(Km)[b]	Strain was obtained directly from the arrayed transposon mutant library. The barcode for PP_2163 was PCR amplified with Barseq_P1 and Barseq_P2 (Tm=55°C, <0.2 kB) and sequence confirmed with BarSeq_SangerSeq.	([11])
RW09	*P. putida* KT2440 *mlaD*::$Tc1_{1101533}$(Km)[b]	Strain was obtained directly from the arrayed transposon mutant library. The barcode for PP_0960 was PCR amplified with Barseq_P1 and Barseq_P2 (Tm=55°C, <0.2 kB) and sequence confirmed with BarSeq_SangerSeq.	([11])
RW217	*P. putida* KT2440 + pTM007	pTM007 was transformed into KT2440. All cultures were grown with 50 mg/L of kanamycin to maintain plasmid.	This study

RW218	*P. putida* KT2440 Δ*oprF* + pTM007	pTM007 was transformed into RW29. All cultures were grown with 50 mg/L of kanamycin to maintain plasmid.	This study
RW219	*P. putida* KT2440 Δ*oprI* + pTM007	pTM007 was transformed into RW30. All cultures were grown with 50 mg/L of kanamycin to maintain plasmid.	This study
RW220	*P. putida* KT2440 + pRW075	pRW075 was transformed into KT2440. All cultures were grown with 50 mg/L of kanamycin to maintain plasmid.	This study
RW221	*P. putida* KT2440 Δ*oprF* + pRW075	pRW075 was transformed into RW29. All cultures were grown with 50 mg/L of kanamycin to maintain plasmid.	This study
RW222	*P. putida* KT2440 Δ*oprI* + pRW075	pRW075 was transformed into RW30. All cultures were grown with 50 mg/L of kanamycin to maintain plasmid.	This study
RW223	*P. putida* KT2440 + pTM008	pTM008 was transformed into KT2440. All cultures were grown with 50 mg/L of kanamycin to maintain plasmid.	This study
RW224	*P. putida* KT2440 Δ*oprF* + pTM008	pTM008 was transformed into RW29. All cultures were grown with 50 mg/L of kanamycin to maintain plasmid.	This study
RW225	*P. putida* KT2440 Δ*oprI* + pTM008	pTM008 was transformed into RW30. All cultures were grown with 50 mg/L of kanamycin to maintain plasmid.	This study
RW226	*P. putida* KT2440 + pBTL-2 (Empty vector)	pBTL-2 empty vector was transformed into KT2440. All cultures were grown with 50 mg/L of kanamycin to maintain plasmid.	This study
RW227	*P. putida* KT2440 Δ*oprF* + pBTL-2 (Empty vector)	pBTL-2 empty vector was transformed into RW29. All cultures were grown with 50 mg/L of kanamycin to maintain plasmid.	This study
RW228	*P. putida* KT2440 Δ*oprI* + pBTL-2 (Empty vector)	pBTL-2 empty vector was transformed into RW30. All cultures were grown with 50 mg/L of kanamycin to maintain plasmid.	This study
AG5577	*P. putida* KT2440 ΔPP_4740::BxB1_RV_phi370_attB cassette ΔPP_4217/4218 intergenic::TG1_BL3_A118_attB cassette ΔPP_2876::R4_phiBT1_MR11_attB cassette	Strain was obtained courtesy of Adam Guss' group at Oak Ridge National Laboratory. The strain contains a total of nine attB attachment sites (3 in each of 3 loci) for use with serine integrase helper plasmids. See reference for more details.	([12])
TM115	AG5577 Δ*oprF*	pRW007 was transformed into AG5577. Deletion of *oprF* (PP_2089) was confirmed by colony PCR with oRW037 and oRW038 (Tm=68°C, 2.3 kB) followed by sequencing by Oxford Nanopore.	This study
TM117	*TM115 with edits at the Bxb1 site* *attL*^{BxB1}::*bla*:*aadA*:P_{tac}:*plsBC*$_{Pp}$*cdsA*$_{Pp}$::*attR*^{BxB1}	TM115 transformed with pTM018 and the corresponding helper plasmid pGW31 targeting the Bxb1 site. Transformants were selected using 100 mg/L spectinomycin. Insertion of the plasmid containing *bla* (amp^r), *aad* (spec^r), and P_{tac}:*plsBC*$_{Pp}$*cdsA*$_{Pp}$ was sequenced confirmed using oTM064_int and oTM65_210 for colony PCR (Tm = 69 °C, 7.75 kB) followed by sequencing by Oxford Nanopore.	This study

TM118	*TM115 with edits at the Bxb1 site* $attL^{BxB1}$::*bla*:*aadA*:P_{tac}:*plsBC*$_{Ec}$*cdsA*$_{Ec}$::$attR^{BxB1}$	TM115 was transformed with pTM017 and the corresponding helper plasmid pGW31 targeting the Bxb1 site. Transformants were selected using 100 mg/L spectinomycin. Insertion of the of the plasmid containing *bla* (amp^r), *aad* ($spec^r$), and P_{tac}:*plsBC*$_{Ec}$*cdsA*$_{Ec}$ was sequenced confirmed using oTM064_int and oTM65_210 for colony PCR (Tm=69°C, 7.75 kB) followed by sequencing by Oxford Nanopore.	This study
TM121	*TM115 with edits to the TG1 site* $attL^{TG1}$::*nptII*:P_{em7}: *accACDB*$_{Ec}$::$attR^{TG1}$	TM115 was transformed with pTM015 and the corresponding helper plasmid pGW38 targeting the TG1 site. Transformants were selected using 50 mg/L of kanamycin. Insertion of the plasmid containing *nptII* (kan^r) and P_{em7}:*accACDB*$_{Ec}$ was sequenced confirmed using oTM064_int and oTM67_419 for colony PCR (Tm=68°C, 6.56 kB) followed by sequencing by Oxford Nanopore.	This study
TM122	*TM115 with edits to the TG1 site* $attL^{TG1}$::*nptII*:P_{tac}:*gpsA*$_{Pp}$:: $attR^{TG1}$	TM115 transformed with pTM045 and the corresponding helper plasmid pGW38 targeting the TG1 site. Transformants were selected using 50 mg/L of kanamycin. Insertion of the plasmid containing *nptII* (kan^r) and P_{tac}:*gpsA*$_{Pp}$ was sequenced confirmed using oTM064_int and oTM67_419 for colony PCR (Tm=68°C, 3.8 kB) followed by sequencing by Oxford Nanopore.	This study
TM123	*TM115 with edits to the TG1 site* $attL^{TG1}$::*nptII*:P_{tac}:*gpsA*$_{Ec}$:: $attR^{TG1}$	TM115 was transformed with pTM044 and the corresponding helper plasmid pGW38 targeting the TG1 site. Transformants were selected using 50 mg/L of kanamycin. Insertion of the plasmid containing *nptII* (kan^r) and P_{tac}:*gpsA*$_{Ec}$ was sequenced confirmed using oTM064_int and oTM67_419 for colony PCR (Tm=68°C, 3.8 kB) followed by sequencing by Oxford Nanopore.	This study
TM124	*TM117 with edits to the TG1 site* $attL^{TG1}$::*nptII*:P_{tac}: *gpsA*$_{Pp}$::$attR^{TG1}$	TM117 was transformed with pTM045 and the corresponding helper plasmid pGW38 targeting the TG1 site. Transformants were selected using 50 mg/L of kanamycin. Insertion of the plasmid containing *nptII* (kan^r) and P_{tac}:*gpsA*$_{Pp}$ was sequenced confirmed using oTM064_int and oTM67_419 for colony PCR (Tm=68°C, 3.8 kB) followed by sequencing by Oxford Nanopore.	This study
TM125	*TM117 with edits to the TG1 site* $attL^{TG1}$::*nptII*:P_{tac}:*gpsA*$_{Ec}$:: $attR^{TG1}$	TM118 transformed with pTM045 and the corresponding helper plasmid pGW38 targeting the TG1 site. Transformants were selected using 50 mg/L of kanamycin. Insertion of the plasmid containing *nptII* (kan^r) and P_{tac}:*gpsA*$_{Ec}$ was sequenced confirmed using oTM064_int and oTM67_419 for colony PCR (Tm=68°C, 3.8 kB) followed by sequencing by Oxford Nanopore.	This study

[a]Numbers following “Tc1” indicate the location of the transposon insertion site (in bp) in the KT2440 genome.

Table S4. Oligonucleotides utilized in this study.

Name	Sequence (5'->3')	Purpose
oRW029	GCACTGCTGAATCTGATGTAG	Colony PCR of pRW005 integration
oRW030	GGACATGACATGGAAGATTC	
oRW033	ATTGTTGTCGGGACGAGATC	Colony PCR of pRW006 integration
oRW034	GAAGTCAATCTCGACTCGG	
oRW037	ATCGGCCTGGAATATTCGGC	Colony PCR of pRW007 integration
oRW038	GACCGGACACTACCCGTAC	
oRW041	GCTTGCAACGTGCCAATGC	Colony PCR of pRW008 integration
oRW042	GGCCAACATCATGGTCGAC	
oRW127	AGCTGGAATACCGCGATGTC	Colony PCR of pRW039 integration
oRW128	CTCCGTTGCATCCCTATCAG	
Barseq_P1	AATGATACGGCGACCACCGAGATCTACACTCTTTCCCTACACGACGCTCTTCCGATCTNNNNNGTCGACCTGCAGCGTACG	Verification of barcode from arrayed transposon library
Barseq_P2	CAAGCAGAAGACGGCATACGAGATCGTGATGTGACTGGAGTTCAGACGTGTGCTCTTCCGATCTGATGTCCACGAGGTCTCT	
BarSeq_SangerSeq	GACCACCGAGATCTACAC	
oTM002	TATCTAGATGGTGAGCAAGGGCGAGG	mNeonGreen was PCR extracted from Addgene Plasmid #182388 and assembled in to pBTL2 using EcoRV and xbaI used to generate pTM002
oTM003	ATGATATCTTAATGATGATGATGATGATGGGTACC	
oTM020	GATATCATTCAGGACGAGCCTCAG	Primers used to PCR pBTL-2 backbone for assembly of pTM007 and pTM008 (Tm 56°C, 2.6 kB).
oTM024	TCCTTAAAATTGAAATAGATTACGGGTGTGAAATTGTTATCCG	
oTM019	GCTCGTCCTGAATGATATCTTAATGATGATGATGATGATGGGTACCTCC	Used to generate VNp_mNeonGreen and mNeonGreen fragments for Gibson assembly into pBTL-2.
oTM022	TTTCAATTTTAAGGAGGTTTTAATAATGGATGTATTCATGAAAGG	
oTM027	TTTCAATTTTAAGGAGGTTTTAATAATGGTGAGCAAGGGCGAGG	
oTM035	CTTCTCTTATAGAGGTTCTAGTGTGAAATTGTTATCCGCTCACAATTCC	Primers used to generate parts for the assembly of pTM018 from *P. putida* KT2440 genome.
oTM036	AGGTAGAAGACAACTGGTCTAGACTCGAGG	
oTM037	CAATTTCACACTAGAACCTCTATAAGAGAAGTTAGCATGACCCGTTCCCCCCTGCACC	
oTM038	AACCTCTTATGTTTTGATGATTAGAGGGTCGAAGTTTCCAGC	
oTM039	GACCCTCTAATCATCAAAACATAAGAGGTTTTTTAATGCTTTATTCATTGCGTATGTTCC	

oTM042	CTCGAGTCTAGACCAGTTGTCTTCTACCTAGTC AAAAGCCTCCGGTCGGAGGCTTTTGACTTCACA TCACACCCCATTCGGCAGC	
oTM043	AAAAAACCTCCTTTAGAGAGAAAGTCAGGCCAG TTCTTTTTCCATGCGGTCAATGC	
oTM044	CCTGACTTTCTCTCTAAAGGAGGTTTTTTCATGC TTAAACAACGCATCATTACTGC	
oTM064_int	TGTTCGTCCTCGAGTCTAGACC	Colony PCR of sage integrase plasmids
oTM065_210	TTCCACTGCCATCAGCGC	
oTM067_419	AGAAGTACTACCAGGGCATTGC	

Table S5. Plasmids utilized in this study.

Name	Description	Construction details	Reference
pRW005	pK18sB-based plasmid for deletion of PP_1121 in *P. putida* KT2440-derived strains	1 kb homology regions upstream and downstream of PP_1121 were designed. An XbaI site was inserted between the two homology regions, and the insert was cloned into the pK18sb backbone at the EcoRI and HindIII sites. The plasmid was synthesized and sequence-verified by Twist Biosciences.	This study
pRW006	pK18sB-based plasmid for deletion of PP_4669 in *P. putida* KT2440-derived strains	1 kb homology regions upstream and downstream of PP_4669 were designed. An XbaI site was inserted between the two homology regions, and the insert was cloned into the pK18sb backbone at the EcoRI and HindIII sites. The plasmid was synthesized and sequence-verified by Twist Biosciences.	This study
pRW007	pK18sB-based plasmid for deletion of PP_2089 (*oprF*) in *P. putida* KT2440-derived strains	1 kb homology regions upstream and downstream of PP_2089 (*oprF*) were designed. An XbaI site was inserted between the two homology regions, and the insert was cloned into the pK18sb backbone at the EcoRI and HindIII sites. The plasmid was synthesized and sequence-verified by Twist Biosciences.	This study
pRW008	pK18sB-based plasmid for deletion of PP_2322 (*oprI*) in *P. putida* KT2440-derived strains	1 kb homology regions upstream and downstream of PP_2322 (*oprI*) were designed. An XbaI site was inserted between the two homology regions, and the insert was cloned into the pK18sb backbone at the EcoRI and HindIII sites. The plasmid was synthesized and sequence-verified by Twist Biosciences.	This study
pRW039	pK18sB-based plasmid for deletion of PP_1502 in *P. putida* KT2440-derived strains	1 kb homology regions upstream and downstream of PP_1502 were designed. An XbaI site was inserted between the two homology regions, and the insert was cloned into the pK18sb backbone at the EcoRI and HindIII sites. The plasmid was synthesized and sequence-verified by Twist Biosciences.	This study
Addgene Plasmid #182390	pRSFDuet-1 based plasmid containing VNp-mNeonGreen_OmpA-mCherry	pRSFDuet-1_VNp-mNeonGreen_OmpA-mCherry was a gift from Dan Mulvihill (Addgene plasmid # 182390 ; http://n2t.net/addgene:182390 ; RRID:Addgene_182390)	[13]
Addgene Plasmid #182388	pRSFDuet-1 based plasmid containing VNp6-mNeonGreen	pRSFDuet-1_VNp6-mNeonGreen was a gift from Dan Mulvihill (Addgene plasmid # 182388 ; http://n2t.net/addgene:182388 ; RRID:Addgene_182388)	([13])
pRW075	pBTL-2-based plasmid for the expression of spycatcher003-	The expression cassette included P_{lac}, ribosome binding site [RBS; translation initiation rate (TIR) = 10277.53], *ompA*$_{EC,}$ linker, spycatcher003, RBS (TIR:	This study

	ompA$_{EC}$:spytag003-mNeonGreen	193180.16), mNeonGreen (originally from Addgene Plasmid #182388), linker, spytag003, and *soxR* terminator. The *ompA*$_{EC,}$ linkers, spycatcher003, and spytag003 were codon optimized using Integrated DNA Technologies Codon Optimization Tool. The RBS was optimized using the Salis optimization tool as described in the methods. The plasmid was synthesized and sequence-verified by Twist Biosciences. The sequence of the expression cassette is listed below.	
pTM001	pRSFDuet based plasmid containing VNp-mNeonGreen with his tag.	Using BglII and NdeI the VNp from Addgene Plasmid #182390 was cloned into the place where VNp was in frame with mNeonGreen in the pRSFDuet vector Addgene Plasmid #182388. Plasmid was sequenced verified by Oxford Nanopore Sequencing.	This study
pTM007	pBTL-2-based plasmid for the expression of mNeonGreen	The expression cassette contained the P_{lac}, RBS (TIR: 193180.16), and mNeonGreen (originally from Addgene Plasmid #182388). This plasmid was assembled by Gibson assembly and fragments were generated using primers oTM020, oTM024, oTM019, and oTM027. The RBS was designed using the Salis optimization tool as described in the methods. The plasmid was sequenced by Oxford Nanopore Sequencing. The sequence of the expression cassette is listed below.	This study
pTM008	pBTL-2-based plasmid for the expression of mNeonGreen-vesicle nucleating peptide (vNP)	The expression cassette contained P_{lac}, RBS (same as above TIR: 305719.82), VNp fused to mNeonGreen (originally from Addgene Plasmid #182388). This plasmid was assembled by Gibson assembly and fragments were generated using primers oTM020, oTM024, oTM019, and oTM022. The RBS was optimized using the Salis optimization tool as described in the methods. The plasmid was sequenced by Oxford Nanopore Sequencing. The sequence of the expression cassette is listed below.	This study
Addgene Plasmid #22806	pBTL-2 plasmid with no insert containing the *lac* promoter	pBTL-2 was obtained courtesy of Ryan Gill (Addgene plasmid # 22806 ; http://n2t.net/addgene:22806; RRID:Addgene_22806).	([14])
pJH0419	Sage integrase plasmid for integration in the BxbI site conferring kanamycin resistance	Plasmid obtained courtesy of Adam Guss' group at Oak Ridge National Laboratory.	([12])
pJH0210	Sage integrase plasmid with the TG1 integration site in the TG1 integration site	Plasmid obtained courtesy of Adam Guss' group at Oak Ridge National Laboratory.	([12])

	conferring ampicillin, spectinomycin, and streptomycin resistance		
pGW31	Integrase Expression Suicide Vector for integration at the Bxb1 site (used for pJH0419)	Plasmid obtained courtesy of Adam Guss' group at Oak Ridge National Laboratory.	([12])
pGW38	Integrase Expression Suicide Vector for integration at the TG1 site (used for pJH0210)	Plasmid obtained courtesy of Adam Guss' group at Oak Ridge National Laboratory.	([12])
pTM015	pJH0210-based plasmid for the expression of $accACDB_{Ec}$	The expression cassette included an operon containing P_{em7}, RBS (TIR: 9830.55), $accA_{Ec}$, RBS (TIR: 11644.13), $accC_{Ec}$, RBS (TIR: 10352.73), $accD_{Ec}$, RBS (TIR: 9318.34), $accB_{Ec}$, and T_{tonB}. This will integrate into the TGI site. The *E. coli accACDB* was codon optimized using Basebuddy with the *P. putida* KT2440 codon usage. The RBS was optimized using the Salis optimization tool as described in the methods. The plasmid was synthesized and sequence-verified by Twist Biosciences. The sequence of the expression cassette is listed below.	This study
pTM017	pJH0419-based plasmid for the expression of $plsBC_{Ec}cdsA_{Ec}$	The expression cassette included an operon containing P_{tac}, RBS (TIR: 12736.66), $plsB_{Ec}$<br>RBS (TIR: 12571.37), $plsC_{Ec}$<br>RBS (TIR: 12845.03), $cdsA_{Ec}$, and T_{tonB}. This will integrate into the Bxb1 site. The *E. coli plsBC* and *cdsA* were codon optimized using Basebuddy with the *P. putida* KT2440 codon usage. The RBS was optimized using the Salis optimization tool as described in the methods. The plasmid was synthesized and sequence-verified by Twist Biosciences. The sequence of the expression cassette is listed below.	This study
pTM018	pJH0419-based plasmid for the expression of $plsBC_{Pp}cdsA_{Pp}$	The expression cassette included an operon containing P_{tac}, RBS (TIR: 12373.93), $plsB_{Pp}$<br>RBS (TIR: 14370.80), $plsC_{Pp}$<br>RBS (TIR: 13749.52), $cdsA_{Pp}$, and T_{tonB}. This will integrate into the Bxb1 site. Genes encoding *plsBC* and *cdsA* were amplified from the *P. putida* KT2440 genome using the primers listed in Table S2. The RBS was optimized using the Salis optimization tool as described in the methods. The plasmid backbone was amplified with oTM035 and oTM036 from pJH419. The plasmid was sequenced verified with Oxford Nanopore. The sequence of the expression cassette is listed below.	This study

pTM044	pJH0210-based plasmid for the expression of *gpsA*$_{Ec}$	The expression cassette included an operon containing P_{tac}, RBS (TIR: 11274.39), *gpsA*$_{Ec}$, and T_{tonB}. This will integrate into the TG1 site. The *E. coli gpsA*$_{Ec}$ was codon optimized using Basebuddy with the *P. putida* KT2440 codon usage. The RBS was optimized using the Salis optimization tool as described in the methods. The plasmid was synthesized and sequence-verified by Twist Biosciences. The sequence of the expression cassette is listed below.	This study
pTM045	pJH0210-based plasmid for the expression of *gpsA*$_{Pp}$	The expression cassette included an operon containing P_{tac}, RBS (TIR: 12388.56), *gpsA*$_{Pp}$, and T_{tonB}. This will integrate into the TG1 site. The native sequence of the *P. putida gpsA*$_{Ec}$ was utilized. The RBS was optimized using the Salis optimization tool as described in the methods. The plasmid was synthesized and sequence-verified by Twist Biosciences. The sequence of the expression cassette is listed below.	This study

Table S6. Sequences for mNeonGreen, tags, and codon-optimized genes. Key: Promoter, **RBS,** gene, linker, tag, and terminator

**Plasmid expression cassette**	**Sequence**
pRW75 - P$_{lac}$:*ompA$_{EC}$*-Spycatcher003:mNeonGreen-Spytag003	TTGCATTAGGCACCCCAGGCTTTACACTTTATGCTTCCGGCTCGTATGTTGT<br>GTGGAATTGTGAGCGGATAACAATTTCACAC**CGCACGATTATTAAGGAGCA**<br>**AATTTTT**ATGAAAAAGACAGCTATCGCGATTGCAGTGGCACTGGCTGGTTTC<br>GCTACCGTAGCGCAGGCCGCTCCGAAAGATAACACCTGGTACACTGGTGCT<br>AAACTGGGCTGGTCCCAGTACCATGACACTGGTTTCATCAACAACAATGGCC<br>CGACCCATGAAAACCAACTGGGCGCTGGTGCTTTTGGTGGTTACCAGGTTA<br>ACCCGTATGTTGGCTTTGAAATGGGTTACGACTGGTTAGGTCGTATGCCGTA<br>CAAAGGCAGCGTTGAAAACGGTGCATACAAAGCTCAGGGCGTTCAACTGAC<br>CGCTAAACTGGGTTACCCAATCACTGACGACCTGGACATCTACACTCGTCTG<br>GGTGGTATGGTATGGCGTGCAGACACTAAATCCAACGTTTATGGTAAAAACC<br>ACGACACCGGCGTTTCTCCGGTCTTCGCTGGCGGTGTTGAGTACGCGATCA<br>CTCCTGAAATCGCTACCCGTCTGGAATACCAGTGGACCAACAACATCGGTGA<br>CGCACACACCATCGGCACTCGTCCGGACAACGGCATGCTGAGCCTGGGTGT<br>TTCCTACCGTTTCGGTGGCGGTGGGGGTTCGGTAACCACCTTATCAGGTTTA<br>TCAGGTGAGCAAGGTCCGTCCGGTGATATGACAACTGAAGAAGATAGTGCT<br>ACCCATATTAAATTCTCAAAACGTGATGAGGACGGCCGTGAGTTAGCTGGTG<br>CAACTATGGAGTTGCGTGATTCATCTGGTAAAACTATTAGTACATGGATTTCA<br>GATGGACATGTGAAGGATTTCTACCTGTATCCAGGAAAATATACATTTGTCGA<br>AACCGCAGCACCAGACGGTTATGAGGTAGCAACTCCAATTGAATTTACAGTT<br>AATGAGGACGGTCAGGTTACTGTAGATGGTGAAGCAACTGAAGGTGACGCT<br>CATACTGGTCACCATCACCATCACCACTAAGATATC**TTTCACACCCGTAATC**<br>**TATTTCAATTTTAAGGAGGTTTTAATA**ATGGTGAGCAAGGGCGAGGAGGATA<br>ACATGGCCTCTCTCCCAGCGACACATGAGTTACACATCTTTGGCTCCATCAA<br>CGGTGTGGACTTTGACATGGTGGGTCAGGGCACCGGCAATCCAAATGATGG<br>TTATGAGGAGTTAAACCTGAAGTCCACCAAGGGTGACCTCCAGTTCTCCCCC<br>TGGATTCTGGTCCCTCATATCGGGTATGGCTTCCATCAGTACCTGCCCTACC<br>CTGACGGGATGTCGCCTTTCCAGGCCGCGATGGTAGATGGCTCCGGATACC<br>AAGTCCATCGCACAATGCAGTTTGAAGATGGTGCCTCCCTTACTGTTAACTA<br>CCGCTACACCTACGAGGGAAGCCACATCAAAGGAGAGGCCCAGGTGAAGG<br>GGACTGGTTTCCCTGCTGACGGTCCTGTGATGACCAACTCGCTGACCGCTG<br>CGGACTGGTGCAGGTCGAAGAAGACTTACCCCAACGACAAAACCATCATCA<br>GTACCTTTAAGTGGAGTTACACCACTGGAAATGGCAAGCGCTACCGGAGCA<br>CTGCGCGGACCACCTACACCTTTGCCAAGCCAATGGCGGCTAACTATCTGA<br>AGAACCAGCCGATGTACGTGTTCCGTAAGACGGAGCTCAAGCACTCCAAGA<br>CCGAGCTCAACTTCAAGGAGTGGCAAAAGGCCTTTACCGATGTGATGGGCA<br>TGGACGAGCTGTACAAGGCTAGCGGAGGAAGCGGAGGTACCCGTGGCGTT<br>CCTCATATTGTTATGGTGGACGCCTACAAACGCTATAAATAAATTCAGGACG<br>AGCCTCAGACTCCAGCGTAACTGGACTGAAAACAAACTAAAGCGCCCTTGTG<br>GCGCTTTAGTTTT
pTM007 - P$_{lac}$:mNeonGreen	TTGCATTAGGCACCCCAGGCTTTACACTTTATGCTTCCGGCTCGTATGTTGT<br>GTGGAATTGTGAGCGGATAACAATTTCACAC**CCGTAATCTATTTCAATTTTAA**<br>**GGAGGTTTTAATA**ATGGTGAGCAAGGGCGAGGAGGATAACATGGCCTCTCT<br>CCCAGCGACACATGAGTTACACATCTTTGGCTCCATCAACGGTGTGGACTTT<br>GACATGGTGGGTCAGGGCACCGGCAATCCAAATGATGGTTATGAGGAGTTA

	AACCTGAAGTCCACCAAGGGTGACCTCCAGTTCTCCCCCTGGATTCTGGTC<br>CCTCATATCGGGTATGGCTTCCATCAGTACCTGCCCTACCCTGACGGGATGT<br>CGCCTTTCCAGGCCGCGATGGTAGATGGCTCCGGATACCAAGTCCATCGCA<br>CAATGCAGTTTGAAGATGGTGCCTCCCTTACTGTTAACTACCGCTACACCTA<br>CGAGGGAAGCCACATCAAAGGAGAGGCCCAGGTGAAGGGGACTGGTTTCC<br>CTGCTGACGGTCCTGTGATGACCAACTCGCTGACCGCTGCGGACTGGTGCA<br>GGTCGAAGAAGACTTACCCCAACGACAAAACCATCATCAGTACCTTTAAGTG<br>GAGTTACACCACTGGAAATGGCAAGCGCTACCGGAGCACTGCGCGGACCAC<br>CTACACCTTTGCCAAGCCAATGGCGGCTAACTATCTGAAGAACCAGCCGATG<br>TACGTGTTCCGTAAGACGGAGCTCAAGCACTCCAAGACCGAGCTCAACTTCA<br>AGGAGTGGCAAAAGGCCTTTACCGATGTGATGGGCATGGACGAGCTGTACA<br>AGGCTAGCGGAGGAAGCGGAGGTACCCATCATCATCATCATCATTAAGATAT<br>CATTCAGGACGAGCCTCAGACTCCAGCGTAACTGGACTGAAAACAAACTAAA<br>GCGCCCTTGTGGCGCTTTAGTTTT
pTM008 - P_{lac}:VNp-mNeonGreen	TTGCATTAGGCACCCCAGGCTTTACACTTTATGCTTCCGGCTCGTATGTTGT<br>GTGGAATTGTGAGCGGATAACAATTTCACAC**CCGTAATCTATTTCAATTTTAA**<br>**GGAGGTTTTAATA**ATGGATGTATTCATGAAAGGACTTTCAAAGGCCAAGGAG<br>GGAGTTGTGGCAGCTGCTGAGAAAACCAAACAGGGTGTGGCAGAAGCAGCA<br>GGAAAGACAAAAGAGGGTGTTCTCAGATCTGGAGGAAGCGGAATGGTGAGC<br>AAGGGCGAGGAGGATAACATGGCCTCTCTCCCAGCGACACATGAGTTACAC<br>ATCTTTGGCTCCATCAACGGTGTGGACTTTGACATGGTGGGTCAGGGCACC<br>GGCAATCCAAATGATGGTTATGAGGAGTTAAACCTGAAGTCCACCAAGGGTG<br>ACCTCCAGTTCTCCCCCTGGATTCTGGTCCCTCATATCGGGTATGGCTTCCA<br>TCAGTACCTGCCCTACCCTGACGGGATGTCGCCTTTCCAGGCCGCGATGGT<br>AGATGGCTCCGGATACCAAGTCCATCGCACAATGCAGTTTGAAGATGGTGC<br>CTCCCTTACTGTTAACTACCGCTACACCTACGAGGGAAGCCACATCAAAGGA<br>GAGGCCCAGGTGAAGGGGACTGGTTTCCCTGCTGACGGTCCTGTGATGACC<br>AACTCGCTGACCGCTGCGGACTGGTGCAGGTCGAAGAAGACTTACCCCAAC<br>GACAAAACCATCATCAGTACCTTTAAGTGGAGTTACACCACTGGAAATGGCA<br>AGCGCTACCGGAGCACTGCGCGGACCACCTACACCTTTGCCAAGCCAATGG<br>CGGCTAACTATCTGAAGAACCAGCCGATGTACGTGTTCCGTAAGACGGAGC<br>TCAAGCACTCCAAGACCGAGCTCAACTTCAAGGAGTGGCAAAAGGCCTTTAC<br>CGATGTGATGGGCATGGACGAGCTGTACAAGGCTAGCGGAGGAAGCGGAG<br>GTACCCATCATCATCATCATCATTAAGATATCATTCAGGACGAGCCTCAGACT<br>CCAGCGTAACTGGACTGAAAACAAACTAAAGCGCCCTTGTGGCGCTTTAGTT<br>TT
pTM044 - P_{tac}:$gpsA_{Ec}$	GAGCTGTTGACAATTAATCATCGGCTCGTATAATGTGTGGAATTGTGAGCGG<br>ATAACAATTTCACAC**CATTAAGATATTTAAGGAAGCTACAAA**ATGAACCAAC<br>GGAACGCCTCAATGACTGTGATCGGTGCAGGCTCGTATGGCACCGCTCTTG<br>CCATCACCCTGGCAAGAAATGGCCACGAGGTTGTCCTCTGGGGCCATGACC<br>CTGAGCATATCGCAACCCTTGAACGCGACCGCTGTAACGCCGCCTTTCTTCC<br>CGATGTGCCTTTCCCGGATACCCTCCACCTTGAAAGCGACCTGGCCACTGC<br>CTTAGCTGCCAGCCGTAATATCCTCGTCGTCGTACCCAGCCATGTCTTTGGG<br>GAGGTGCTGCGCCAGATTAAGCCCTTGATGCGTCCTGATGCGCGTCTGGTG<br>TGGGCCACAAAAGGGCTAGAAGCAGAAACCGGCCGTTTGTTACAGGACGTG<br>GCCCGAGAGGCCTTAGGCGATCAAATTCCGCTGGCGGTAATCTCTGGCCCA<br>ACGTTTGCGAAAGAACTGGCCGCAGGTTTACCGACAGCCATTTCGCTGGCC<br>TCGACCGATCAGACCTTTGCCGATGATCTGCAGCAGCTGCTCCACTGCGGC<br>AAGAGTTTCCGCGTTTACAGCAATCCGGATTTCATTGGCGTGCAGTTGGGCG<br>GCGCGGTGAAAAACGTTATTGCCATTGGTGCGGGGATGTCCGACGGTATAG<br>GTTTTGGTGCCAATGCGAGGACGGCCCTGATAACCCGTGGATTGGCCGAAA

	TGTCCAGACTTGGTGCGGCGCTTGGTGCCGATCCAGCTACCTTTATGGGCA<br>TGGCTGGACTTGGCGATCTGGTGCTGACCTGTACCGACAACCAGTCGCGTA<br>ACCGCCGTTTTGGCATGATGCTGGGTCAGGGCATGGATGTACAGAGCGCGC<br>AGGAGAAGATTGGCCAGGTGGTGGAAGGCTACCGCAATACGAAGGAAGTCC<br>GCGAACTGGCGCACCGCTTTGGCGTTGAGATGCCAATCACCGAGGAGATCT<br>ACCAAGTGTTGTACTGCGGGAAGAACGCTCGCGAAGCAGCATTGACCTTAC<br>TGGGTCGAGCACGCAAGGACGAGCGCAGCAGCCACTGAAGTCAAAAGCCT<br>CCGACCGGAGGCTTTTGACT
pTM045 -<br>$P_{tac:}gpsA_{Pp}$	GAGCTGTTGACAATTAATCATCGGCTCGTATAATGTGTGGAATTGTGAGCGG<br>ATAACAATTTCACAC**TAAGCGAGTCAACATAAGGGGGTACGGG**ATGACTGA<br>ACAGCAACCTGTTGCGGTTCTGGGCGGCGGCAGCTTCGGCACCGCCGTGG<br>CAAACCTGCTGGCGGAAAACGGTGTGCCGGTGCGCCAATGGATGCGCGAC<br>CCGGCGCAGGCCGAGGCCATGCGTGTGAATCGCGAGAATCCGCGCTATCT<br>CAAGGGTATCCGCCTGCACGATGGGGTTGAGCCGGTCAACGACTTGCTGGC<br>CACCCTGCAGGCCAGCGAGCTGATCTTCGTTGCCCTGCCATCGAGTGCCTT<br>GCGCAGCGTGCTGGCACCGCATGCCGAGCTGCTGCGCGGCAAGGGCCTG<br>GTCAGCCTGACCAAGGGCATCGAAGCGCAAAGCTTCAAGCTGATGAGCCAG<br>ATCCTCGAAGAGATCGCCCCCGAGGCGCGCATCGGCGTTTTGTCGGGGCC<br>CAACCTGGCGCGCGAAATCGCCGAACATGCGCTGACTGCCACAGTGGTGG<br>CCAGTGAGCACGAAGACCTGTGCCAGCAGGTACAGGCCGTACTGCACGGG<br>CGCACCTTCCGCGTCTACGCCAGTGCCGACCGTTTCGGCGTCGAACTGGGC<br>GGTGCACTGAAGAACGTCTACGCGATCATCGCCGGCATGGCGGTGGCCTTG<br>GGGATGGGCGAGAACACCAAGAGCATGCTGATTACCCGGGCCTTGGCCGA<br>GATGACCCGTTTCGCCGTCAGCCAGGGCGCCAACCCCATGACGTTCCTCGG<br>CCTGGCCGGTGTCGGTGACCTGATCGTCACCTGCTCCTCGCCCAAGAGCCG<br>CAACTATCAGGTTGGCTACGCACTGGGGCAGGGCCAAAGCCTGGAAGAGG<br>CGGTCAACCGCCTGGGCGAAGTGGCCGAGGGGGTCAACACGCTCAAGGTG<br>CTCAAGACCAAGGCCCAGCAAGTGCAGGTGTACATGCCGCTGGTGGCTGG<br>CCTGCATGCCATCCTGTTCGAAGGGCGCACGCTGAACCAGGTGATCGAGCA<br>CCTGATGCGCGCCGAGCCCAAGACCGATGTCGATTTCATTTCCATCAGCGG<br>TTTCAACTGAAGTCAAAAGCCTCCGACCGGAGGCTTTTGACT
pTM017 -<br>$P_{tac:}plsBC_{Ec}cdsA_{Ec}$	TTGACAATTAATCATCGGCTCGTATAATGTGTGGAATTGTGAGCGGATAACA<br>ATTTCACAC**TCAACCCTTTTAAGGAGGTCTTG**ATGTCGGGCTGGCCACGAAT<br>TTATTACAAATTGCTGAATCTGCCATTGTCCATCCTGGTCAAAAGCAAGTCGA<br>TTCCGGCCGATCCAGCCCCGGAACTGGGGTTGGACACCTCACGTCCAATCA<br>TGTACGTCTTGCCGTACAACTCGAAAGCCGACCTCTTGACGTTGCGCGCCC<br>AGTGCTTGGCGCATGACTTGCCAGACCCGTTAGAGCCGTTGGAAATCGATG<br>GCACGCTCTTGCCTCGGTACGTGTTTATCCACGGCGGGCCGCGTGTGTTCA<br>CCTATTACACGCCGAAAGAAGAGAGTATCAAACTGTTTCACGACTATTTGGA<br>CTTGCACCGTAGCAACCCAAATCTGGATGTGCAGATGGTGCCAGTGTCGGT<br>GATGTTTGGTAGGGCTCCTGGGCGTGAGAAAGGCGAAGTGAACCCGCCGC<br>TGCGAATGTTGAACGGCGTCCAGAAGTTTTTCGCGGTCTTGTGGTTGGGTC<br>GCGACTCGTTCGTGCGGTTCTCGCCGTCAGTTTCGCTGCGCCGTATGGCCG<br>ACGAACACGGCACGGACAAAACGATAGCACAGAAACTGGCCCGAGTGGCC<br>CGTATGCACTTTGCCCGCCAACGCTTAGCAGCCGTAGGCCCAAGGTTGCCT<br>GCTCGTCAGGACCTGTTCAATAAGCTGCTAGCCTCCAGGGCCATTGCCAAA<br>GCGGTAGAAGATGAAGCGCGCTCCAAAAAAATCTCGCATGAAAAAGCCCAA<br>CAGAACGCGATCGCACTGATGGAAGAAATCGCGGCGAATTTCTCCTACGAA<br>ATGATCCGCTTGACTGACCGTATTCTGGGCTTCACCTGGAACCGACTTTACC<br>AGGGCATAAACGTCCATAACGCAGAGCGCGTTCGCCAGTTGGCCCACGACG<br>GGCATGAGCTGGTCTATGTGCCATGCCACCGCTCCCACATGGACTACCTGC<br>TGTTGTCGTACGTGCTATACCACCAGGGTTTGGTGCCTCCGCATATCGCAGC<br>CGGTATCAACCTCAATTTCTGGCCTGCGGGGCCGATTTTCCGCCGCCTGGG<br>TGCCTTCTTCATTAGGCGTACGTTCAAAGGCAACAAACTCTATTCGACCGTTT

	TCCGGGAATACCTCGGCGAACTGTTCAGCCGCGGTTACTCGGTCGAGTACT<br>TCGTGGAAGGCGGGCGGTCCAGGACTGGTCGACTACTGGACCCGAAAACT<br>GGTACGTTGTCGATGACCATCCAGGCCATGTTGCGTGGCGGTACGCGCCCG<br>ATTACGTTGATCCCGATCTACATCGGCTACGAACATGTCATGGAAGTGGGCA<br>CTTACGCCAAAGAACTGCGAGGCGCCACCAAAGAGAAGGAAAGCCTGCCGC<br>AGATGCTGCGCGGCTTAAGCAAACTGCGTAACTTGGGTCAGGGCTACGTCA<br>ACTTCGGTGAACCAATGCCGCTTATGACGTACTTGAACCAGCATGTCCCAGA<br>CTGGCGCGAATCAATCGACCCTATCGAAGCCGTCCGTCCTGCATGGTTGAC<br>GCCGACGGTCAACAATATTGCAGCCGATCTCATGGTCCGCATTAACAACGCA<br>GGAGCGGCAAACGCCATGAACCTGTGCTGCACTGCGCTTCTGGCATCACGT<br>CAGCGCTCATTGACCCGCGAGCAATTGACCGAACAACTTAACTGCTACCTG<br>GATCTGATGCGAAATGTGCCTTACTCCACGGACTCCACCGTCCCATCCGCCT<br>CAGCCAGCGAACTTATCGACCACGCCCTTCAAATGAACAAGTTCGAAGTCGA<br>AAAAGACACGATCGGCGACATCATCATTCTGCCTAGGGAACAAGCCGTGCT<br>CATGACCTACTACCGCAACAACATCGCGCACATGTTGGTGCTTCCTTCGCTA<br>ATGGCGGCAATCGTGACCCAGCACCGGCACATCTCGCGCGACGTCCTTATG<br>GAACACGTCAATGTGTTGTACCCAATGCTGAAAGCGGAGCTGTTCCTGCGAT<br>GGGATCGCGACGAATTGCCGGACGTCATTGATGCGCTGGCAAATGAGATGC<br>AACGGCAGGGTCTGATCACCCTGCAAGATGACGAGTTGCATATTAACCCGG<br>CCCATAGTAGGACGCTTCAGCTGTTGGCCGCAGGTGCGCGCGAAACGCTG<br>CAACGCTATGCCATCACCTTCTGGTTGTTGAGTGCCAACCCGTCGATCAACA<br>GGGGGACGCTGGAGAAAGAAAGCAGAACCGTCGCCCAACGTCTCTCGGTG<br>TTGCACGGCATCAACGCGCCGGAGTTCTTCGACAAGGCGGTGTTCTCGTCG<br>CTGGTGCTGACGCTGCGTGACGAGGGTTATATCAGCGATAGCGGCGATGCC<br>GAACCGGCAGAAACGATGAAAGTCTATCAGTTGCTGGCCGAATTGATCACTT<br>CCGACGTGCGTTTGACGATCGAATCCGCGACGCAGGGCGAAGGTTGA**TCG**<br>**CCACTACCTAGGAGGTGCCCA**AATGCTTTACATCTTCCGCTTGATCATCACC<br>GTGATCTACTCGATCTTGGTCTGCGTATTCGGCTCGATTTACTGCTTGTTCA<br>GCCCGCGCAACCCGAAACACGTGGCCACCTTTGGTCACATGTTCGGTCGCT<br>TGGCCCCGCTGTTCGGCCTGAAAGTCGAATGCCGCAAACCAACGGACGCG<br>GAAAGCTACGGCAACGCGATCTACATCGCGAACCACCAGAACAACTACGAC<br>ATGGTGACTGCGTCGAACATCGTGCAACCGCCGACGGTGACGGTCGGCAAA<br>AAGAGCTTGCTGTGGATACCTTTCTTCGGTCAGTTGTACTGGTTGACCGGCA<br>ACTTGTTGATCGACAGGAACAACCGCACGAAAGCGCACGGCACCATCGCGG<br>AAGTCGTGAACCACTTCAAAAAACGCCGCATCTCCATCTGGATGTTCCCGGA<br>AGGTACCCGCAGCCGTGGTCGCGGTCTGCTTCCGTTCAAAACGGGTGCATT<br>CCACGCGGCAATTGCGGCCGGCGTCCCGATTATTCCTGTGTGCGTCTCGAC<br>TACGTCGAATAAAATCAACTTGAACCGACTGCACAACGGCCTGGTGATCGTC<br>GAAATGCTGCCGCCAATCGACGTCTCGCAGTATGGCAAAGACCAGGTCCGC<br>GAACTGGCGGCCCACTGCCGTTCGATAATGGAACAAAAAATCGCCGAATTG<br>GACAAAGAAGTCGCAGAACGCGAAGCCGCCGGGAAAGTCTGA**CCAACGTT**<br>**CCTGAGGAGTCCCAAAC**ATGCTGAAATACCGCCTGATATCGGCGTTCGTGT<br>TGATACCTGTCGTCATCGCCGCCTTGTTTCTGCTCCCGCCGGTGGGTTTCG<br>CCATTGTCACGCTCGTGGTCTGCATGTTGGCAGCCTGGGAATGGGGGCAGT<br>TGAGCGGTTTCACGACGCGCTCGCAGAGGGTATGGTTGGCCGTGTTGTGCG<br>GTTTATTGTTGGCCTTGATGCTCTTCCTGTTGCCGGAATATCACCGAAACATC<br>CATCAACCGCTGGTCGAAATCTCCTTGTGGGCATCGCTGGGCTGGTGGATC<br>GTCGCCCTTTTGCTGGTGCTGTTCTACCCAGGTTCGGCGGCAATCTGGCGT<br>AACTCGAAAACGTTGCGCTTGATCTTCGGCGTGCTTACCATCGTCCCTTTCT<br>TCTGGGGCATGCTGGCCCTCCGGGCCTGGCACTATGACGAAAACCACTACT<br>CGGGCGCGATATGGCTGTTGTACGTCATGATCCTGGTATGGGGCGCGGACT<br>CCGGCGCGTACATGTTCGGCAAATTGTTCGGTAAACACAAACTGGCACCGA

	AGGTGTCGCCGGGCAAAACCTGGCAAGGCTTCATCGGTGGGCTCGCAACT<br>GCAGCGGTAATCTCGTGGGGCTACGGCATGTGGGCCAATTTGGACGTCGCA<br>CCTGTCACCTTGTTGATCTGCTCGATTGTCGCGGCCTTGGCCTCCGTGCTC<br>GGCGATCTGACCGAATCGATGTTCAAACGCGAAGCGGGGATCAAAGACAGC<br>GGCCACTTGATTCCAGGGCACGGTGGCATCCTCGACCGCATTGATAGCCTG<br>ACGGCTGCCGTACCGGTCTTTGCATGCTTGTTGCTTCTGGTATTCAGGACGT<br>TGTGAAGTCAAAAGCCTCCGACCGGAGGCTTTTGACT
pTM018 -<br>P_{tac}:*plsBC*$_{Pp}$*cdsA*$_{Pp}$	TTGACAATTAATCATCGGCTCGTATAATGTGTGGAATTGTGAGCGGATAACA<br>ATTTCACAC**TAGAACCTCTATAAGAGAAGTTAGC**ATGACCCGTTCCCCCCT<br>GCACCGCCTGATATTCGGCGGCCTGCGTCGCCTGTTGTACCTGTGGGTGCG<br>CTCCGAGACCATCAACCAGTCTTCCATGACCCTCAACCTCGACCGCAGCCG<br>GCCGGTGTTCTATGCGTTGCCCTCGCCCGCGCTCACCGACCTGGCCGTGCT<br>CGACCACGAGTGCACCAAGGCGGGGCTGCCGCGCCCGGTGCTGCCGGTA<br>GCCGTCGGCCCACTGCAGGAACCCGCCGCGTTCTTCTACCTGACCCCCGAC<br>CCGGACTGGCTCGGCCGCCAGGACAAAAGCGGCGCCCCGCCCACGCTCGA<br>GCGCCTGGTGGCTGCCGTCAGCCAGCATGCCGAAGAAGATGCGCAGATCA<br>TTCCTGTCAGCGTGTTCTGGGGCCAGACCCCGGCCAGCGAGTCCAGCCCCT<br>GGAAGCTGCTGTTCGCCGACAGCTGGGCGGTAACCGGGCGCCTGCGCCGG<br>CTGCTGACCGTACTGATCCTGGGGCGCAAGACCCGGGTACAGTTCTCCGCG<br>CCAATCCACCTGCGCGAACTGGTGCAGCACAACAAAGGCCACGAGCGCACC<br>GTGCGCATGGCCCAGCGCCTGATGCGCGTGCACTTTCGCAACCTCAAGACT<br>GCCGTCATCGGCCCGGACATCTCGCACCGGCGCACCCTGGTCAAAGGCCT<br>GGTCCATGCCCCGCAAGTGCGCCAGGCGATTGCCGACGAAGCTCAGCGCG<br>AGAACCTGCCGCTGGCCAAAGCCGAGGCCCAGGCACTGCGCTATGGCAAC<br>GAGATAGCCTCGGACTACACCTATACCGCCATCCGCTTCCTCGAAGTGGTG<br>CTCAGCTGGTTCTGGAACAAGATCTACGACGGCATCAAGGTCAACCACATTG<br>AACAGGTGCAGGGTATCGCCCCTGGCCATGAAGTGATCTACGTGCCATGCC<br>ACCGCAGCCACATCGACTACCTGCTGCTGTCGTACCTGCTGTTCCGCAACG<br>GCCTCACCCCGCCGCACGTGGCTGCAGGCATCAACCTCAACATGCCGGTG<br>GTCGGTAACCTGCTACGCCGTGGTGGCGCCTTCTTCATGCGCCGCACGTTC<br>AAGGGCAACCCGCTTTACACGGCAGTGTTCAACGAGTACCTGCACACCCTG<br>TACACCAAGGGCTTCCCGGTCGAGTACTTCGTCGAAGGCGGCCGCTCGCG<br>CACCGGGCGCATGTTGCAGCCGCGCACCGGGATGCTGGCCATTACCCTGC<br>GCAGCTTCCTGCGTTCGTCGCGTACGCCAATCGTGTTCGTGCCGGTGTACA<br>TCGGCTACGAGCGCGTGCTCGAAGGCCGTACCTACCTGGGCGAACTGCGC<br>GGCGCCAGCAAGAAGAAGGAGTCGGTGTTGGACATCTTCAAGGTGTTCGGC<br>GCACTCAAGCAGCGCTTTGGCCAGGTCTACGTCAACTTCGGCGAACCCATC<br>CGCCTGGCTGGTTTCCTCGACCAGCAGCAGCCCGGCTGGCGAGAACAGGA<br>TCACGGTCCGCAGTACCGACCGGAATGGCTCAACGCCACCACCGCCCGCC<br>TGGGAGAAACCGTGGCCCGCCACCTCAACGAGGCGGCCGCCATCAACCCG<br>GTCAACCTGGTGGCCCTGGCACTGCTGTCCACCAGCCGCCTTGCCCTGGAC<br>GAGCGCGCCCTGACCCGCGTACTCGACCTGTACCTGGCGCTGCTGCGCCA<br>AGTGCCCTACTCGCCGCACACCACCCTGCCCGAGGGCGACGGCCAGGCAC<br>TGATCGAACATGTGCGCAGCATGAACCTGGTGGCCGAGCAGAAGGACGCC<br>CTGGGCCGCATCCTCTACCTGGATGAAGGCAACGCGGTGCTGATGACCTAC<br>TACCGCAACAACGTGCTGCACATCTTCGCCCTGCCGGCGCTGCTGGCCAGC<br>TTCTTCCTCAGCAGTTCCCGCATGAGCCGCCAACTGCTGGGCCAGTATGTG<br>CATGCGCTTTATCCCTATCTGCAGGCCGAACTGTTCCTGCGCTGGACGCCA<br>GAACAGCTGGACGAGGTCATCGACCAATGGCTGGTCGCGCTGGTGGAACA<br>GGGCCTGCTGCGCCAGGACAACGACCTGTACGTGCGCCCGGCGCCCAGCT<br>CGCGGCAGTTCGTGTTGCTGACCCTGCTCGCCCGCACCATCACCCAGACCC

	TGCAGCGCTTCTACATGGCAACCTCGCTGCTGATCAACAGCGGGCAGAACA<br>GCCTGAGCGCCGAAGCGCTGGAAGACCTGTGCGTGATGATGGCCCAGCGC<br>CTGTCGATCCTGCATGGCCTGAACGCACCGGAGTTCTTCGACAAGACGCTG<br>TTCCGCCACTTCATTCAGACCTTGCTTCAGCAAGGCGTGCTGCACGCCGAC<br>GCGCAAGGCAAGCTGAGTTATCACGACAAGCTCGGCGAGCTGGCCGAGGG<br>CGTGGCCAAGCGAGTACTGTCGGCCGAGCTGCGCCTGTCGATCCGCCAGG<br>TGGCCCTGCACCGTGACGACGGGCTGGAAACTTCGACCCTCTAAT**TCATCAA**<br>**AACATAAGAGGTTTTTTA**ATGCTTTATTCATTGCGTATGTTCCTGCTGGGGC<br>TGCATTTTCTTGCCGTTGGCGCCGTGGGCCTGCTCATTGGCCTGTGCCGCC<br>CCTTCAACCCTGACAACAGCCGCGTTTTCGCCCGGCTCTACAGCTTGCCGG<br>CCACCTGGCTGATGCGCATCGAGGTCAAGGCCGAAGTCGGCCCATTGTGG<br>GACCACCCGCCCGGCTGCGTGATCGTGGCCAACCACCAGTCCAACTTCGAT<br>CTGTTCGTGCTGGGCCAAGTGGTGCCGCAGCGCACCGTCGCCATCGGCAA<br>GAAGAGCCTGGGCTGGATCCCGCTGTTCGGCCAACTGTTCTGGCTGGGCG<br>GCAACGTGCTGGTCGACCGCAAGAATGCCTATCAGGCGCGCAGGGCCTTAC<br>AGAAGACCACCCGGGTTCTGCAGGACGACACCTCGATCTGGATTTTCCCCG<br>AAGGCACCCGCAATCCCGGCGAGCATCTGCTGGCGTTCAAGAAAGGCGCAT<br>TTCACATGGCCATCGAGGCCGGTGTGCCGATCGTGCCGGTCTGCGTCAGCC<br>GCTATGCCAGGCGCCTGAGCCTGAACAGCTGGCGCCAGCGCACGGTGATT<br>GTGCGCTCGCTGCCGCCCATTGCCACGACGGGCATGACGCTGCAGGACCT<br>GCCAGCGCTGATCGAGCAATGCCGTGGGCAAATGCAGCAGTGCATTGACCG<br>CATGGAAAAAGAACTGGCCTGA**CTTTCTCTCTAAAGGAGGTTTTTTC**ATGCT<br>TAAACAACGCATCATTACTGCGCTGATCCTGCTGCCGGTCGCGCTGGGTGG<br>TTTCTTCCTGCTCAATGGCGGGGATTTCGCCCTGTTCATCGGCTTCGTAGTG<br>ACCCTCGGTGCCTGGGAGTGGGCGCGCCTTGCCGGGCTGATGGCCCAGCC<br>GCTGCGCATTGCCTATGCAGCGGTGGTCGCGGGGGCGCTGATGCTGCTGC<br>ACATCCTTCCGGAACTGGCGCCCTGGGTGCTGGGCGCTGCCGTGATCTGGT<br>GGGGGCTGGCCACCTGGCTGGTGCTTACCTACCCGCGCAGCAGCGACCTG<br>TGGGCCAGTGCGGCCTGTCGGTTGTTGATCGGCCTGCTGGTGTTGCTGCCG<br>GCCTGGCAAGGGCTGGTGCTGCTCAAGCACTGGCCCTTGGGCAACTGGCT<br>GATCCTGTCGGTCATGGTGCTGGTGTGGGCCGCCGACATCGGTGCGTACTT<br>CTCTGGCCGGGCATTCGGCAAGCGCAAGCTGGCTCCGCAGGTCAGCCCAG<br>GCAAGAGCTGGGAAGGCGTGTACGGTGGTCTGGCGGTCAGCCTGTTGATTA<br>CCCTGGGTGTCGGCATCAGCCGCGACTGGGGCTTTGGTCAGATCCTGCTG<br>GGCCTGTTGGGCGCTGCGTTGCTGGTGATGTCCTCGGTGGTCGGTGACCT<br>GACCGAGAGCATGTTCAAGCGTCGCTCCGGCATCAAGGACAGCAGCAATCT<br>GCTGCCCGGGCATGGGGGTGTGCTCGATCGCATTGACAGCCTGACTGCCG<br>CAATCCCGATCTTCGCCGTGCTGTTGTGGGCTGCCGAATGGGGTGTGATGT<br>GAAGTCAAAAGCCTCCGACCGGAGGCTTTTGACT
pTM015 -<br>P_{em7}:*accACDB*$_{Ec}$	TTAATTAATTGTTGACAATTAATCATCGGCATAGTATATCGGCATAGTATAATA<br>CGACAAGGTGAGGAACTAAACCCCTAGG**GGTCCTCACCTAAGGAGGTCCC**<br>**CAA**ATGTCGCTGAACTTCTTGGACTTCGAACAGCCGATCGCGGAACTGGAA<br>GCCAAAATCGACTCGCTGACGGCCGTGAGCCGCCAGGACGAAAAACTGGA<br>CATCAACATCGACGAAGAAGTGCACCGCCTGCGCGAAAAAAGCGTCGAACT<br>GACGCGCAAAATCTTCGCCGACCTTGGCGCGTGGCAGATTGCCCAACTGGC<br>ACGCCATCCACAGCGCCCATACACCCTGGACTACGTCCGCCTGGCGTTCGA<br>CGAATTCGACGAACTGGCAGGCGACCGCGCCTACGCGGACGACAAAGCAA<br>TCGTCGGTGGTATCGCCCGCTTGGACGGTCGCCCGGTGATGATCATCGGCC<br>ACCAAAAAGGCCGCGAAACCAAAGAAAAAATCCGCCGCAACTTCGGCATGC<br>CAGCCCCAGAAGGCTACCGCAAAGCACTGCGTCTGATGCAAATGGCGGAAC<br>GCTTCAAAATGCCAATCATCACCTTCATCGACACCCCGGGTGCGTACCCAG

	GCGTGGGTGCAGAAGAACGCGGTCAGAGTGAAGCCATCGCGCGCAACCTG<br>CGTGAAATGTCGCGCTTGGGCGTCCCGGTCGTCTGCACGGTCATCGGTGAA<br>GGTGGTAGTGGCGGTGCCCTGGCCATTGGCGTGGGCGACAAAGTGAACAT<br>GTTGCAATACAGCACCTACTCGGTCATCTCGCCGGAAGGCTGTGCCTCGAT<br>TCTGTGGAAAAGCGCCGACAAAGCCCCGCTGGCCGCGGAAGCAATGGGCA<br>TCATTGCACCGCGCCTGAAAGAACTGAAACTGATCGACTCGATCATCCCGGA<br>ACCACTGGGCGGTGCTCACCGTAACCCGGAAGCCATGGCCGCATCGTTGAA<br>AGCCCAACTGCTGGCGGACCTGGCCGACTTGGACGTGTTGAGCACGGAAG<br>ATTTGAAAAACCGCCGCTACCAGCGCCTGATGAGCTACGGCTACGCCTGA**A**<br>**CATGATTGGTAAGGAAAAACAGAG**ATGCTGGACAAAATCGTCATCGCCAAC<br>CGCGGCGAAATCGCGTTGCGTATCTTGCGCGCCTGCAAAGAACTGGGCATC<br>AAAACGGTCGCAGTGCACTCCAGCGCCGACCGCGACCTTAAACACGTCTTG<br>CTGGCGGACGAAACGGTCTGCATCGGCCCAGCGCCGTCCGTCAAATCGTAC<br>CTGAACATCCCGGCGATCATCAGCGCCGCGGAAATCACCGGTGCGGTCGC<br>GATCCATCCGGGTTACGGCTTCTTGTCGGAAAACGCCAACTTCGCCGAACA<br>GGTCGAACGCTCGGGCTTCATCTTCATCGGCCCGAAAGCGGAAACCATCCG<br>CCTGATGGGCGACAAAGTCTCGGCGATCGCGGCGATGAAAAAAGCCGGCG<br>TGCCATGCGTACCGGGTAGTGATGGCCCGCTGGGCGACGATATGGACAAAA<br>ACCGCGCCATCGCGAAACGCATCGGTTACCCGGTGATAATCAAAGCCAGTG<br>GCGGTGGTGGTGGTCGAGGTATGCGCGTAGTCCGCGGTGATGCGGAACTG<br>GCGCAATCGATCTCGATGACCCGCGCCGAAGCCAAAGCCGCGTTCAGCAAC<br>GACATGGTCTACATGGAAAAATACCTGGAAAACCCACGCCACGTCGAAATCC<br>AGGTCCTGGCAGACGGCCAGGGCAACGCAATCTACCTGGCCGAACGCGAC<br>TGCTCGATGCAACGCCGCCACCAGAAAGTGGTCGAAGAAGCCCCAGCACC<br>GGGCATTACCCCGGAACTGCGTCGCTACATCGGCGAACGTTGCGCGAAAGC<br>CTGCGTCGACATCGGCTACCGCGGTGCAGGCACGTTCGAATTCCTGTTCGA<br>AAACGGCGAATTCTACTTCATCGAAATGAACACCCGCATCCAGGTAGAACAC<br>CCGGTCACGGAAATGATCACCGGCGTCGACCTGATCAAAGAACAGCTGAGG<br>ATCGCCGCCGGCCAACCGCTGTCGATCAAACAAGAAGAAGTCCACGTCCGC<br>GGCCACGCCGTGGAATGCCGCATCAACGCCGAAGATCCGAACACCTTCCTG<br>CCATCGCCGGGCAAAATCACCCGCTTCCACGCGCCAGGCGGTTTTGGCGTC<br>CGTTGGGAATCGCACATCTACGCCGGCTACACCGTCCCGCCGTACTACGAC<br>TCCATGATCGGCAAACTGATCTGCTACGGCGAAAACCGTGACGTGGCCATC<br>GCCCGCATGAAAAACGCCTTGCAGGAACTGATCATCGACGGTATCAAAACC<br>AACGTCGATCTGCAGATACGCATCATGAACGACGAAAACTTCCAGCACGGC<br>GGCACGAACATCCACTACCTGGAGAAAAAATTGGGCCTGCAGGAAAAATGA<br>**ACGGCACATTTAAGGAGCCCTGACC**ATGAGCTGGATCGAACGAATCAAAAG<br>CAACATCACGCCTACCCGCAAAGCCAGCATCCCAGAAGGTGTGTGGACTAA<br>ATGCGACAGCTGTGGCCAGGTCTTGTACCGCGCGGAACTGGAACGCAACTT<br>GGAAGTCTGCCCGAAATGCGACCACCACATGCGCATGACGGCCCGCAACC<br>GCCTGCACAGCCTGTTGGACGAAGGTAGCTTGGTGGAACTGGGTAGCGAAT<br>TGGAGCCGAAAGACGTGCTGAAATTCCGCGACTCGAAAAAATACAAAGACC<br>GCCTGGCGTCGGCCCAGAAAGAAACCGGCGAAAAAGACGCCCTGGTGGTG<br>ATGAAAGGCACGCTGTACGGGATGCCGGTCGTCGCCGCCGCATTCGAATTC<br>GCCTTCATGGGCGGCTCCATGGGTTCGGTCGTGGGTGCACGTTTCGTGCGT<br>GCCGTAGAACAGGCCCTGGAAGATAACTGCCCGCTGATCTGCTTCTCGGCC<br>TCGGGCGGCGCACGTATGCAGGAAGCACTGATGTCGCTGATGCAGATGGC<br>CAAAACCTCGGCCGCGCTGGCGAAAATGCAGGAACGCGGCTTGCCGTACAT<br>CTCGGTGTTGACCGACCCGACGATGGGCGGCGTCAGTGCAAGTTTCGCCAT<br>GCTGGGCGACTTGAACATCGCGGAACCGAAAGCCTTGATCGGCTTCGCCGG<br>CCCGCGCGTCATCGAACAGACCGTACGCGAAAAACTGCCGCCAGGGTTCCA<br>GCGCTCGGAATTCCTGATCGAAAAAGGCGCCATCGACATGATCGTCCGCCG

	CCCGGAAATGCGCCTGAAACTGGCCAGCATTCTGGCCAAATTGATGAACCT<br>GCCAGCCCCGAATCCAGAAGCCCCGCGTGAAGGCGTAGTAGTACCACCGG<br>TCCCGGACCAGGAACCAGAAGCCTAA**AGTGAGAACGCTAGGAGTGAGAGG**<br>**T**ATGGACATCCGCAAAATCAAAAAACTGATCGAACTGGTCGAAGAATCCGGC<br>ATCTCGGAACTGGAAATCTCGGAAGGCGAAGAATCGGTCCGCATCAGCCGT<br>GCAGCACCAGCCGCAAGTTTCCCAGTGATGCAACAAGCCTACGCGGCGCCA<br>ATGATGCAGCAGCCAGCACAAAGTAACGCAGCCGCACCAGCAACTGTACCA<br>TCGATGGAAGCGCCAGCAGCAGCAGAAATCAGCGGCCACATCGTCCGTTCG<br>CCGATGGTAGGTACTTTCTACCGCACCCCAAGCCCGGACGCGAAAGCCTTC<br>ATCGAAGTGGGCCAGAAAGTCAACGTGGGCGACACCCTGTGCATCGTCGAA<br>GCCATGAAAATGATGAACCAGATCGAAGCCGACAAATCGGGCACCGTGAAA<br>GCGATCCTGGTCGAATCGGGGCAACCGGTCGAATTTGACGAACCGCTGGTC<br>GTCATCGAATGAAGTCAAAAGCCTCCGACCGGAGGCTTTTGACT

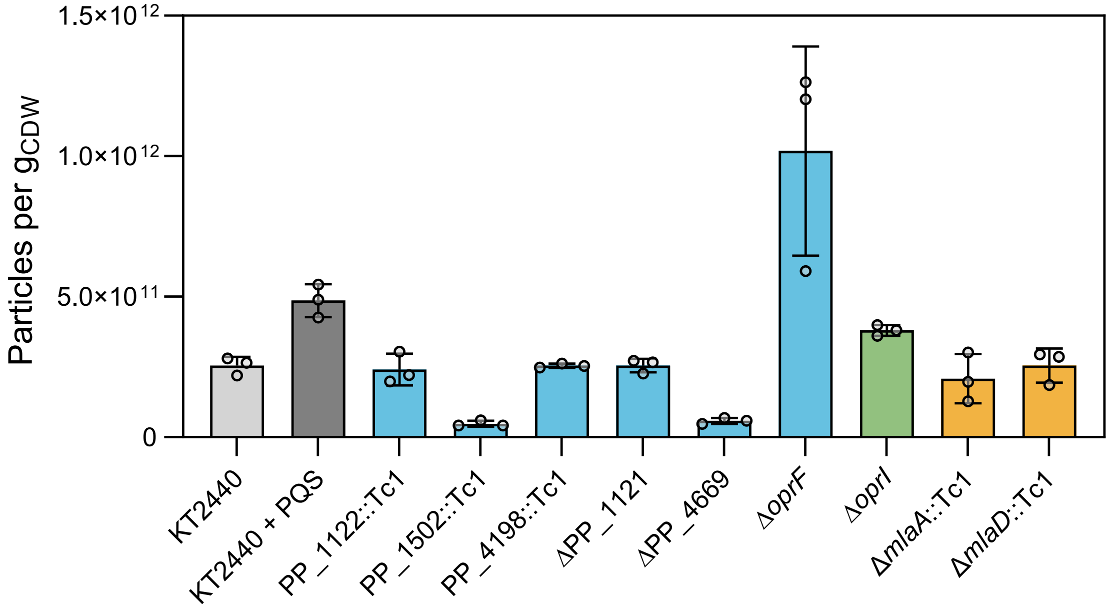

Figure S1. Particle count per g_{CDW} for KT2440 and knockout strains corresponding to data presented in **Fig. 1B.** The absolute counts are provided in **Excel file 1.** The data represent the mean ± the standard deviation determined from three biological replicates. Individual points are illustrated for each biological replicate.

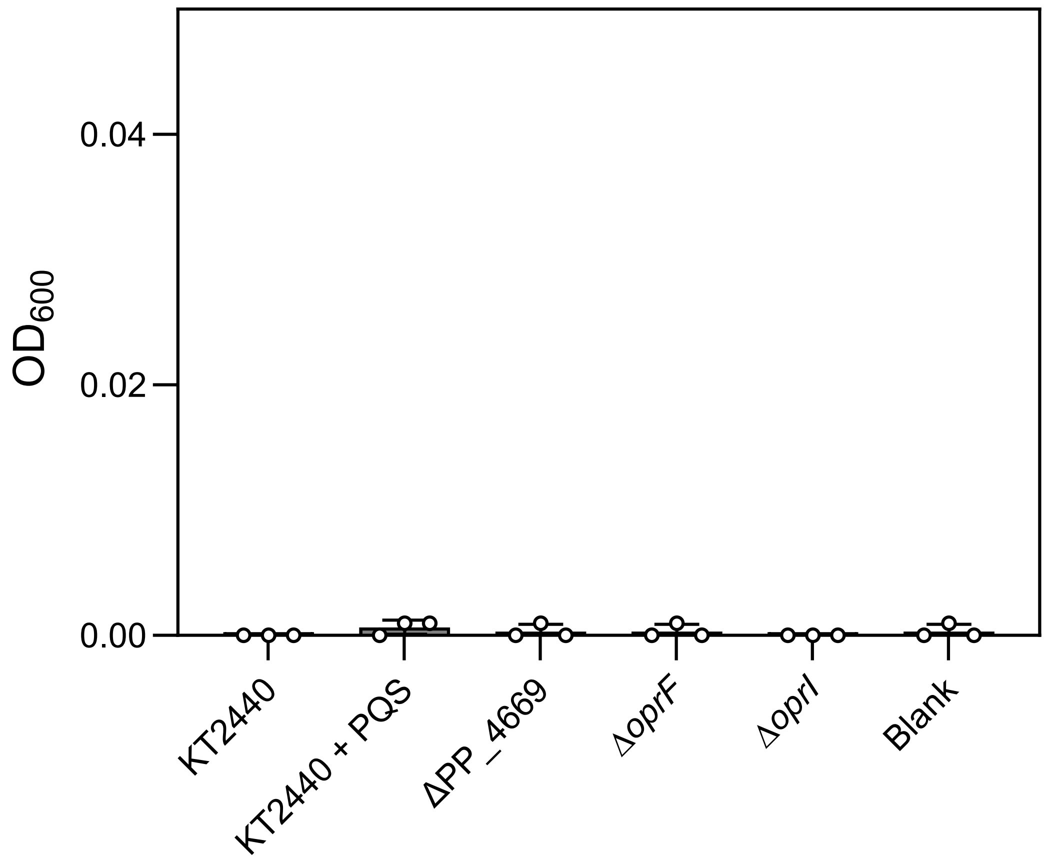

Figure S2. OD_{600} measurements of extracted MVs from KT2440 and knockout strains. The blank consisted of ultrapure MiliQ water and was not significantly different than any of the MV extracts. The data represent the mean ± the standard deviation determined from three biological replicates. Individual points are illustrated for each biological replicate. Significant differences (p < 0.05) were determined with an unpaired two-tailed *t*-test between the strains and the blank.

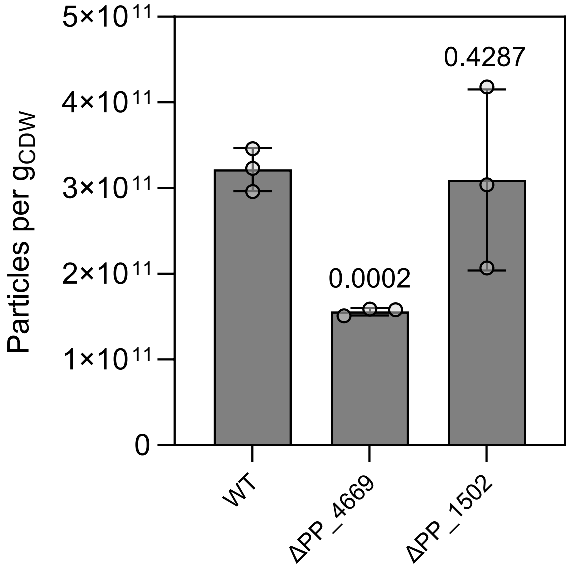

Figure S3. Particle count per g_{CDW} for WT, ΔPP_4669, and ΔPP_1502 grown on 20 mM glucose. The absolute counts are provided in **Excel file 1.** Significant differences (p < 0.05) were calculated using unpaired one-tailed *t*-test to test the hypothesis that the deletion strains decreased MVs production compared to WT. The data represent the mean ± the standard deviation determined from three biological replicates. Individual points are illustrated for each biological replicate.

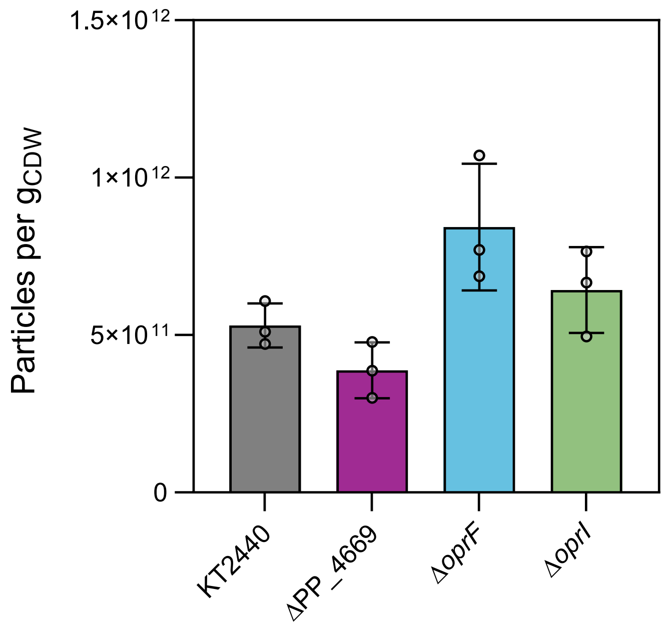

Figure S4. Particle count per g_{CDW} for KT2440 and knockout strains corresponding to data presented in **Fig. 3C.** The absolute counts are provided in **Excel file 1.** The data represent the mean ± the standard deviation determined from three biological replicates. Individual points are illustrated for each biological replicate.

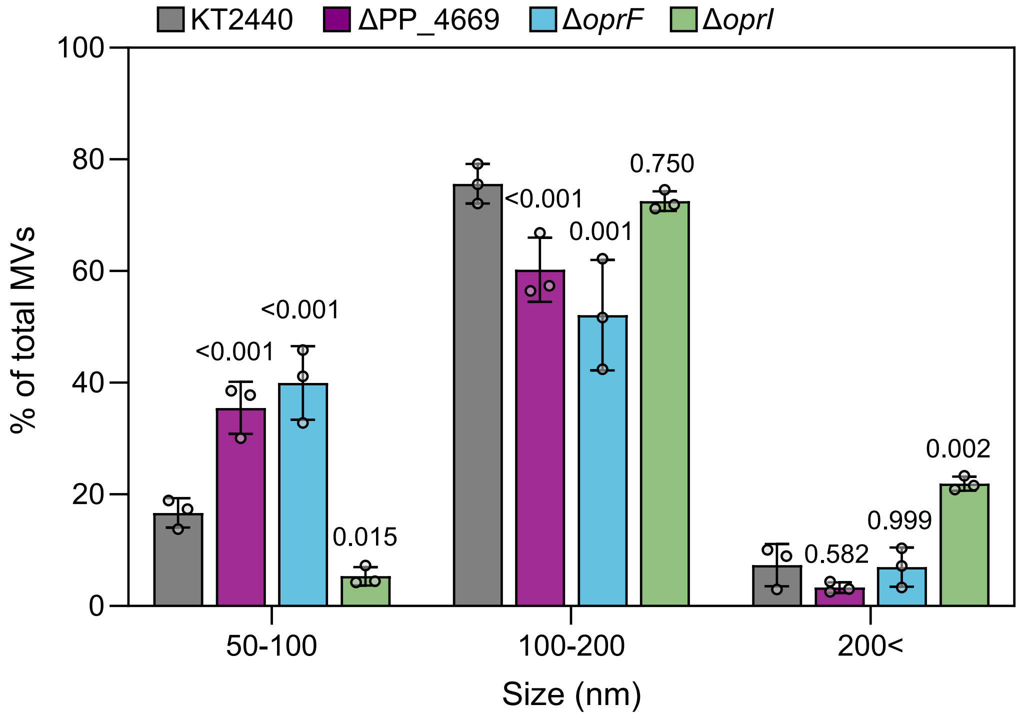

Figure S5. Sizes of MVs corresponding to particle counts in **Fig. S3** were binned into three size ranges and presented as a percentage of the total MV population. The data represent the mean ± the standard deviation determined from three biological replicates. Individual points are illustrated for each biological replicate. Exact *p*-values were determined by two-way ANOVA followed by Dunnett's multiple comparisons test to determine significant differences between the deletion strains and KT2440 in each size range.

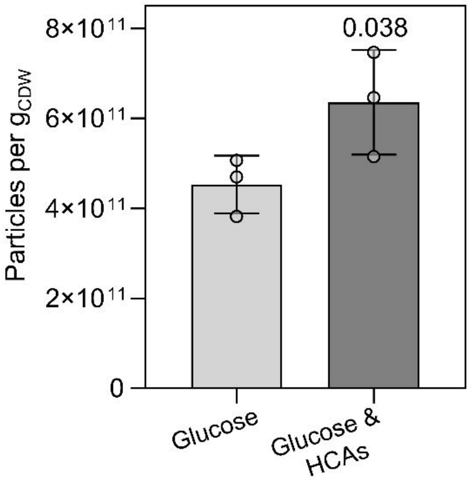

Figure S6. Particle count per g_{CDW} for KT2440 grown on 20 mM glucose alone or 20 mM glucose plus 12.5 mM p-coumarate and 12.5 mM ferulate. The absolute counts are provided in **Excel file 1.** The data represent the mean ± the standard deviation determined from three biological replicates. Individual points are illustrated for each biological replicate.

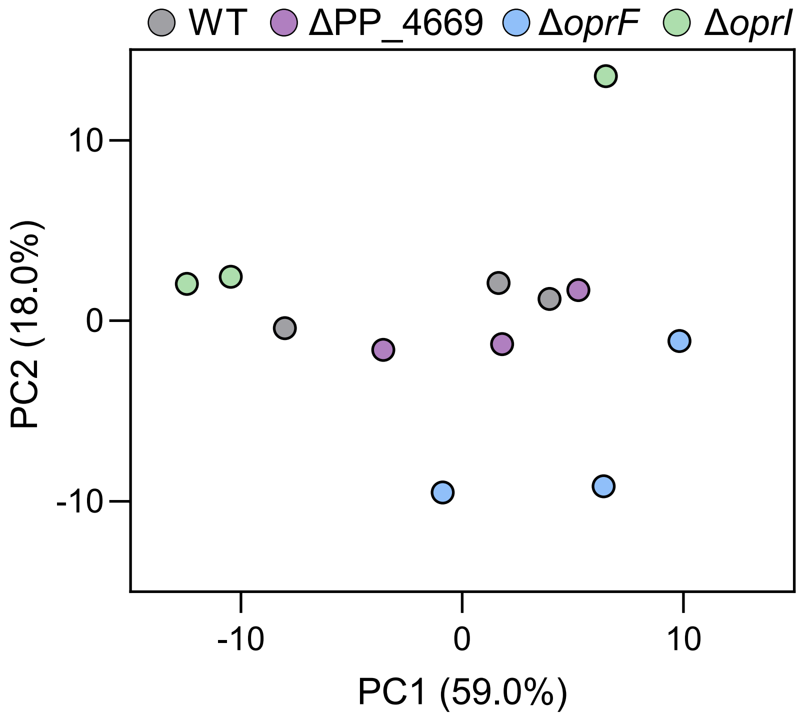

Figure S7. Principal component analysis of the cellular fractions**.**

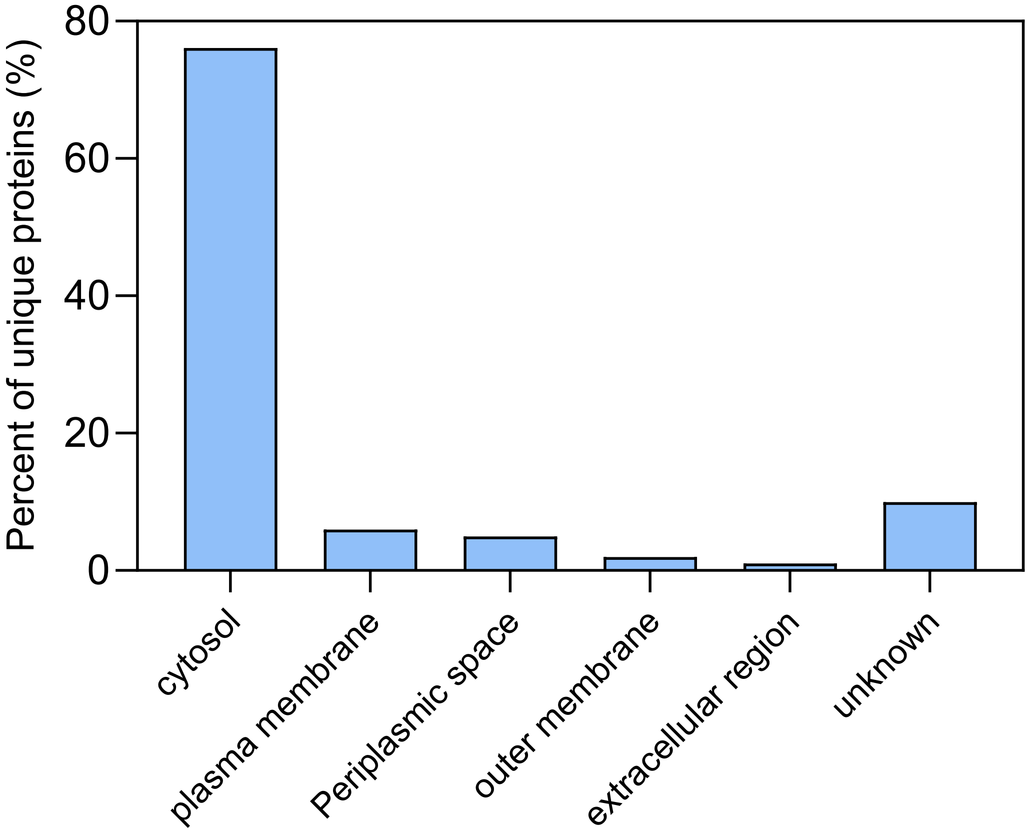

Figure S8. The percentage of proteins classified in a specific cellular location relative to the total number (201) of unique proteins found in the MV fraction for Δ*oprF* compared to the KT2440, Δ*oprI*, and ΔPP_4669. These proteins correspond to the 201 unique proteins found only in the MV fraction for Δ*oprF* illustrated in **Fig. 4E.**

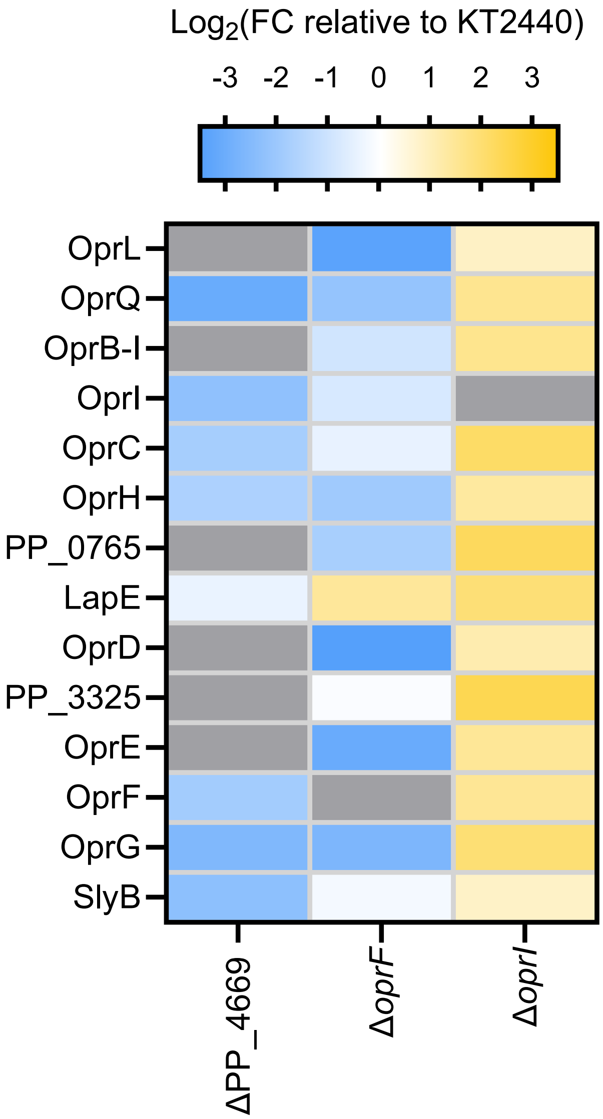

Figure S9. Heatmap of outer membrane proteins in MV fraction with differential abundance, or fold change (FC), for the knockout strains relative to KT2440. Data represents the mean of three biological replicates.

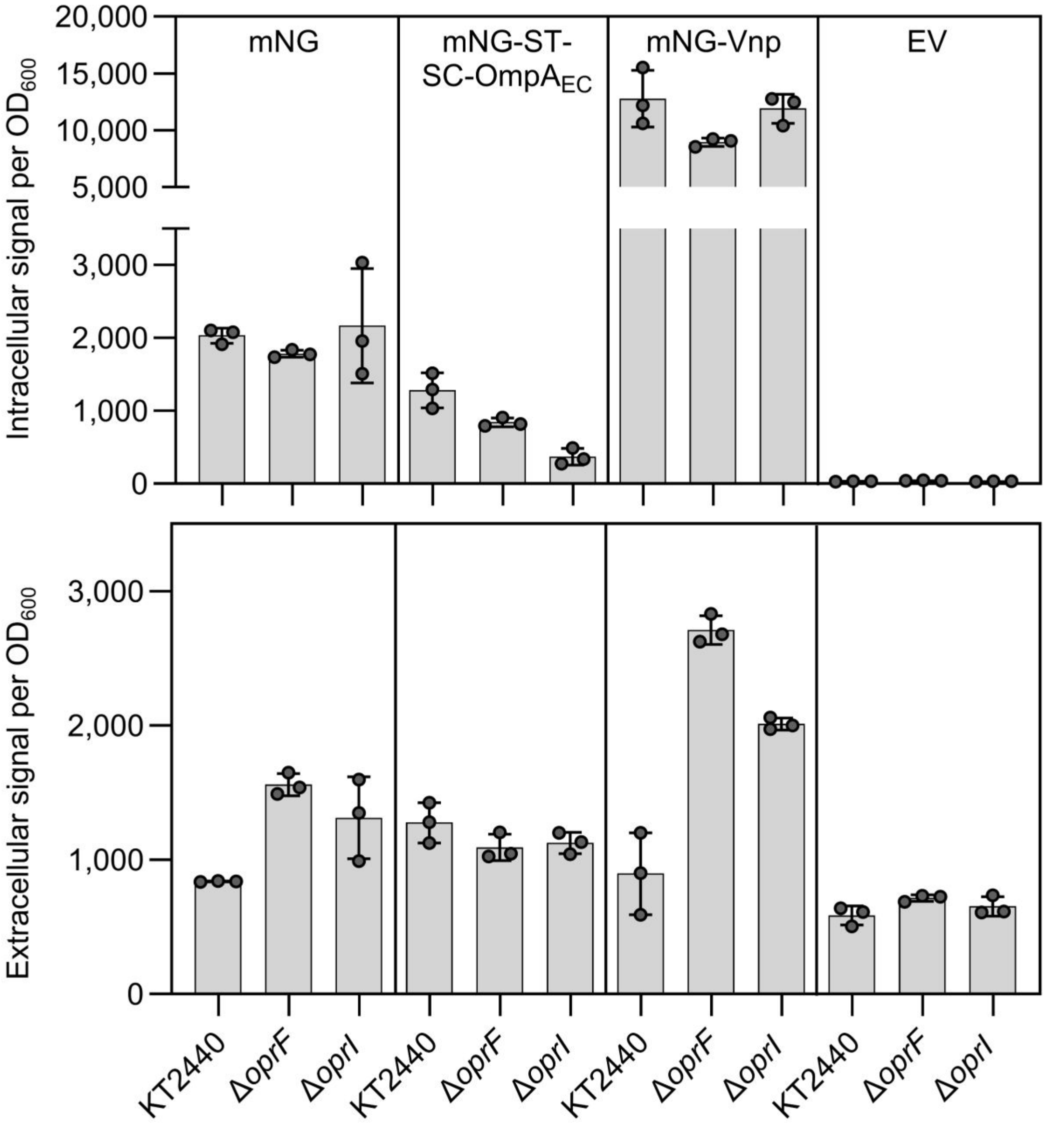

Figure S10. mNeonGreen (mNG) fluorescence signal for the cellular fraction and the extracellular fraction normalized by the OD600 of the cell suspension. The data represent the mean ± the standard deviation determined from three biological replicates. Individual points are illustrated for each biological replicate. Abbreviations include: ST, Spytag; SC, Spycatcher; EV, empty vector.

REFERENCES

(1) Deatherage, B. L.; Lara, J. C.; Bergsbaken, T.; Barrett, S. L. R.; Lara, S.; Cookson, B. T. Biogenesis of Bacterial Membrane Vesicles. *Mol Microbiol* **2009**, *72* (6), 1395–1407.

(2) Song, J.W.; Baeg, Y.; Jeong, H.Y.; Lee, J.; Oh, D.-K.; Hollmann, F.; Park, J.B. Bacterial Outer Membrane Vesicles as Nano-Scale Bioreactors: A Fatty Acid Conversion Case Study. *ChemCatChem* **2021**, *13*, 4080–4086.

(3) McBroom, A. J.; Kuehn, M. J. Release of Outer Membrane Vesicles by Gram-Negative Bacteria Is a Novel Envelope Stress Response. *Mol Microbiol* **2007**, *63* (2), 545–558.

(4) Nevermann, J.; Silva, A.; Otero, C.; Oyarzún, D. P.; Barrera, B.; Gil, F.; Calderón, I. L.; Fuentes, J. A. Identification of Genes Involved in Biogenesis of Outer Membrane Vesicles (OMVs) in *Salmonella Enterica* Serovar Typhi. *Front Microbiol* **2019**, *10* (FEB).

(5) Moon, D. C.; Choi, C. H.; Lee, J. H.; Choi, C. W.; Kim, H. Y.; Park, J. S.; Kim, S. Il; Lee, J. C. *Acinetobacter baumannii* Outer Membrane Protein a Modulates the Biogenesis of Outer Membrane Vesicles. *Journal of Microbiology* **2012**, *50* (1), 155–160.

(6) Wessel, A. K.; Liew, J.; Kwon, T.; Marcotte, E. M.; Whiteley, M. Role of *Pseudomonas aeruginosa* Peptidoglycan-Associated Outer Membrane Proteins in Vesicle Formation. *J Bacteriol* **2013**, *195* (2), 213–219.

(7) Bitzenhofer, N. L.; Höfel, C.; Thies, S.; Weiler, A. J.; Eberlein, C.; Heipieper, H. J.; Batra-Safferling, R.; Sundermeyer, P.; Heidler, T.; Sachse, C.; Busche, T.; Kalinowski, J.; Belthle, T.; Drepper, T.; Jaeger, K. E.; Loeschcke, A. Exploring Engineered Vesiculation by *Pseudomonas putida* KT2440 for Natural Product Biosynthesis. *Microb Biotechnol* **2024**, *17* (1), e14312.

(8) Ojima, Y.; Sawabe, T.; Konami, K.; Azuma, M. Construction of Hypervesiculation *Escherichia coli* Strains and Application for Secretory Protein Production. *Biotechnol Bioeng* **2020**, *117*, 701–709.

(9) Roier, S.; Zing, F. G.; Cakar, F.; Durakovic, S.; Kohl, P.; Eichmann, T. O.; Klug, L.; Gadermaier, B.; Weinzerl, K.; Prassl, R.; Lass, A.; Daum, G.; Reidl, J.; Feldman, M. F.; Schild, S. A Novel Mechanism for the Biogenesis of Outer Membrane Vesicles in Gram-Negative Bacteria. *Nat Commun* **2016**, *7*, 10515.

(10) Grasekamp, K. P.; Beaud Benyahia, B.; Taib, N.; Audrain, B.; Bardiaux, B.; Rossez, Y.; Izadi-Pruneyre, N.; Lejeune, M.; Trivelli, X.; Chouit, Z.; Guerardel, Y.; Ghigo, J. M.; Gribaldo, S.; Beloin, C. The Mla System of Diderm Firmicute *Veillonella parvula* Reveals an Ancestral Transenvelope Bridge for Phospholipid Trafficking. *Nat Commun* **2023**, *14* (1), 1–15.

(11) Rand, J. M.; Pisithkul, T.; Clark, R. L.; Thiede, J. M.; Mehrer, C. R.; Agnew, D. E.; Campbell, C. E.; Markley, A. L.; Price, M. N.; Ray, J.; Wetmore, K. M.; Suh, Y.; Arkin, A. P.; Deutschbauer, A. M.; Amador-Noguez, D.; Pfleger, B. F. A Metabolic Pathway for Catabolizing Levulinic Acid in Bacteria. *Nat Microbiol* **2017**, *2* (12), 1624–1634.

(12) Elmore, J. R.; Furches, A.; Wolff, G. N.; Gorday, K.; Guss, A. M. Development of a High Efficiency Integration System and Promoter Library for Rapid Modification of *Pseudomonas putida* KT2440. *Metab Eng Commun* **2017**, *5*, 1–8.

(13) Eastwood, T. A.; Baker, K.; Streather, B. R.; Allen, N.; Wang, L.; Botchway, S. W.; Brown, I. R.; Hiscock, J. R.; Lennon, C.; Mulvihill, D. P. High-Yield Vesicle-Packaged Recombinant Protein Production from *E. coli*. *Cell Reports Methods* **2023**, *3* (2), 100396.

(14) Lynch, M. D.; Gill, R. T. Broad Host Range Vectors for Stable Genomic Library Construction. *Biotechnol Bioeng* **2006**, *94* (1), 151–158.
